# Proteomic Validation of MEG-01-Derived Extracellular Vesicles as Representative Models for Megakaryocyte- and Platelet-Derived Extracellular Vesicles

**DOI:** 10.3390/biom15121698

**Published:** 2025-12-05

**Authors:** Jose Manuel Sanchez-Manas, Sonia Perales, Gonzalo Martinez-Navajas, Jorge Ceron-Hernandez, Cristina M. Lopez, Angela Peralbo-Molina, Juan R. Delgado, Joaquina Martinez-Galan, Veronica Ramos-Mejia, Eduardo Chicano-Galvez, Maria Hernandez-Valladares, Francisco M. Ortuno, Carolina Torres, Pedro J. Real

**Affiliations:** 1 Gene Regulation, Stem Cells and Development Group, PTS, Granada GENyO, Pfizer-University of Granada-Andalusian Regional Government Centre for Genomics and Oncological Research, Avenida de la Ilustración 114, 18016 Granada, Spain; jose.sanchez@genyo.es (J.M.S.-M.); sopero@ugr.es (S.P.); gm24@sanger.ac.uk (G.M.-N.); jorge96on@gmail.com (J.C.-H.); veronica.ramos@genyo.es (V.R.-M.); 2 Department of Biochemistry and Molecular Biology I, Faculty of Science, University of Granada, Avenida Fuentenueva s/n, 18071 Granada, Spain; 3 Instituto de Investigación Biosanitaria ibs. GRANADA, 18012 Granada, Spain; juanramondelgado@gmail.com (J.R.D.); jmgalan22@hotmail.com (J.M.-G.); mariahv@ugr.es (M.H.-V.); 4 Liquid Biopsies and Cancer Interception Group, PTS, Ganada GENyO, Pfizer-University of Granada-Andalusian Regional Government Centre for Genomics and Oncological Research, Avenida de la Ilustración 114, 18016 Granada, Spain; 5 IMIBIC Mass Spectrometry and Molecular Imaging Unit, Maimonides, Biomedical Research Institute of Cordoba (IMIBIC), Reina Sofia University Hospital, University of Córdoba, Avenida Menéndez Pidal s/n, 14004 Cordoba, Spain; cristina.lopez@imibic.org (C.M.L.); angela.peralbo@imibic.org (A.P.-M.); eduardo.chicano@imibic.org (E.C.-G.); 6 Department of Medical Oncology, Virgen de las Nieves University Hospital, Avenida de las Fuerzas Armadas 2, 18014 Granada, Spain; 7 Department of Cellular Biology, Faculty of Science, University of Granada, Avenida Fuentenueva s/n, 18071 Granada, Spain; 8 Department of Physical Chemistry, Faculty of Science, University of Granada, Avenida Fuentenueva s/n, 18071 Granada, Spain; 9 Department of Computer Engineering, Automatics and Robotics, C.I.T.I.C., University of Granada, Calle Periodista Rafael Gómez Montero 2, 18014 Granada, Spain; fortuno@ugr.es; 10 Department of Biochemistry and Molecular Biology III and Immunology, Faculty of Medicine, University of Granada, Av. Doctor Jesús Candel Fábregas 11, 18016 Granada, Spain

**Keywords:** MEG-01 cell line, extracellular vesicles (EVs), platelets, proteomics, microvesicles (MVs), exosomes (EXOs)

## Abstract

Platelets and their extracellular vesicles (EVs) have emerged as promising liquid biopsy biosources for cancer detection and monitoring. The megakaryoblastic MEG-01 cell line offers a controlled system for generating platelet-like particles (PLPs) and EVs through valproic-acid-induced differentiation. Here, we performed comprehensive characterization and proteomic validation of MEG-01-derived populations, native human platelets, and their EVs using nanoparticle tracking analysis, transmission electron microscopy, imaging flow cytometry and quantitative proteomics. MEG-01 megakaryocytic differentiation is characterized by polylobulated nuclei, proplatelet formation, and elevated CD41/CD42a expression. PLPs predominantly exhibit an activated-like phenotype (CD62P+, degranulated morphology), while microvesicles (100–500 nm) and exosomes (50–250 nm) displayed size distributions and phenotypic markers consistent with native platelet-derived EVs. Proteomics identified substantial core proteomes shared across fractions and fraction-specific patterns consistent with selective cargo partitioning during EV biogenesis. Functional enrichment indicated that MEG-01-derived vesicles preserve key hemostatic, cytoskeletal, and immune pathways commonly associated with platelet EV biology. Ingenuity Pathway Analysis showed that PLPs exhibit proliferative transcriptional programs (elevated MYC/RB1/TEAD1, reduced GATA1), while plasma exosomes display minimal differential pathway activation compared to MEG-01 exosomes. Overall, these findings suggest that MEG-01-derived EVs approximate certain aspects of megakaryocyte-lineage exosomes and activated platelet-like states, although they do not fully replicate native platelet biology. Notably, plasma exosomes show strong proteomic convergence with MEG-01 exosomes, whereas platelet exosomes retain distinct activation-related features.

## 1. Introduction

Contemporary biomedical research increasingly focuses on liquid biopsy as a minimally invasive and highly informative source for the diagnosis and monitoring of complex diseases, including cancer and inflammatory pathologies [1]. Within this context, platelets and their derivatives, particularly extracellular vesicles (EVs), including microvesicles (MVs) and exosomes (EXOs), have emerged as bio-sources of critical importance [2].

Platelets are not merely effectors of hemostasis; they are dynamic enucleated cells that also modulate immune response and tumor progression. Their highly conserved molecular cargo, encapsulated within EVs, reflects the physiological state of their parent cell (the megakaryocyte) and is modified following interaction with the pathological microenvironment (e.g., tumor-educated platelets, TEPs) [3,4]. The capacity of platelet EVs to transfer their protein, lipid, and nucleic acid cargo to target cells positions them as key vehicles in intercellular communication and as accessible reservoirs for the development of liquid biopsies [5,6].

Despite their promise, reliance on platelets and EVs derived from healthy donors introduces a significant methodological challenge: the intrinsic heterogeneity of the sample. Factors such as donor health status, blood processing, and genetic variability contribute to the inconsistency of molecular profiles in EVs, hindering standardization and reproducibility in large-scale functional and biomarker analyses [7,8]. This variability underscores the need for in vitro cellular models that enable the controlled and scalable production of EVs with a representative molecular and biological composition.

The in vitro production of megakaryocytes and functional platelets relies primarily on two human stem cell sources, each presenting a distinct balance of advantages and methodological hurdles [9,10]. First, Human CD34^+^ hematopoietic stem cells (HSCs) isolated from diverse sources such as bone marrow, peripheral blood, or cord blood can be efficiently differentiated into mature megakaryocytes and platelets using both in vivo and in vitro approaches, with protocols ranging from cytokine-driven culture systems to advanced 3D co-culture methods that mimic the bone marrow microenvironment [11,12]. Second, approaches utilizing human pluripotent stem cells (hPSCs) have successfully employed forward programming with transcription factors (e.g., GATA1, FLI1, GATA2, and TAL1) to enable scalable production of mature megakaryocytes and platelets [13,14,15] or the generation of immortalized progenitor cell lines yielding functional platelets [16,17].

Despite these advancements, the field faces significant challenges that limit the utility and scalability of-stem cell-derived products. Key hurdles include the complexity and lack of standardization across differentiation protocols, resulting in high costs and low production efficiency. Furthermore, these systems are constrained by the need for precise transcription factor manipulation and the often limited quantitative evidence provided to ensure the functional equivalence of the resulting platelets to those derived in vivo.

In this context, the MEG-01 cell line, an immortalized human megakaryoblastic line, presents itself as a tractable and convenient alternative. Culturing MEG-01 cells provides a robust, scalable, and economical production system, capable of releasing platelet-like particles (PLPs) and EVs under strictly controlled culture conditions. Despite its demonstrated utility in preliminary functional studies, its immortalized nature raises the critical question of whether MEG-01-derived EVs (MEG-01 EVs) are molecularly and biologically equivalent to their counterparts derived from primary human megakaryocytes and platelets.

The main objective of this study was to assess the extent to which MEG-01 cell-line-derived EVs recapitulate key molecular features of megakaryocyte- and platelet-derived EVs. To address this, we performed a comprehensive comparative proteomic analysis of MEG-01 EVs and EVs isolated from primary platelets. Our results reveal substantial concordance in core signaling pathways and functional markers, supporting the use of MEG-01-derived EVs as a standardized and scalable surrogate model for selected aspects of platelet EV biology, while acknowledging that they do not fully replicate native platelet properties.

## 2. Materials and Methods

### 2.1. Cell Lines and Culture Conditions

MEG-01 is a megakaryoblastic cell line that serves as an in vitro precursor of PLPs [18]. The MEG-01 cell line was cultured in RPMI 1640 medium (BioWest, Nuaille, France) supplemented with 10% fetal bovine serum (FBS) and 1% penicillin-streptomycin (P/S) (Biowest). Human embryonic kidney (HEK293T) cells were grown in Dulbecco’s Modified Eagle Medium (DMEM) (Biowest) supplemented with 10% FBS, without antibiotics. Both cell lines were obtained from the American Type Culture Collection (ATCC, Manassas, VA, USA), maintained at 37 °C in a humidified 5% CO_2_ atmosphere, and used during their exponential growth phase. Periodically mycoplasma testing and short tandem repeat (STR) DNA profiling were performed to confirm cell line authenticity and prevent cross-contamination or phenotypic drift. Human platelets from healthy donors served as controls in selected experiments.

For EV isolation experiments, RPMI 1640 medium was supplemented with exosome-depleted FBS (EXOD-FBS), prepared by ultracentrifugation at 100,000× *g* for 18 h at 4 °C under vacuum (Beckman Coulter Optima XL, 70 Ti rotor, Nyon, Switzerland) followed by double filtration through 0.22 µm PVDF membrane (MILLEX^®^VV Durapore^®^, Merck Millipore, Darmstadt, Germany) to eliminate residual EVs and prevent contamination. This procedure eliminated 70.93% of the total extracellular vesicles present in FBS, as quantified by nanoparticle tracking analysis and consistent with previous findings [19].

### 2.2. Reagents

A 2 mM measure of valproic acid (VPA), a histone deacetylase (HDAC) previously reported to induce megakaryocytic differentiation and stimulate PLP production in MEG-01 cells, was used in this study [19]. A 0.3 M VPA stock solution was prepared in Milli-Q water and stored at −20 °C. Prostaglandin E1 (PGE1) was dissolved in 2.82 mL DMSO to yield a 1000 µM solution. Tyrode’s buffer consisted of 134 mM NaCl, 12 mM NaHCO_3_, 2.9 mM KCl, 0.34 mM Na_2_HPO_4_, 1 mM MgCl_2_, and 10 mM HEPES (pH 7.4). The 100× activation buffer contained 100 mM CaCl_2_, 20 U/mL thrombin, and 1 mg/mL collagen. All these reagents were acquired from Sigma-Aldrich/Merck (St. Louis, MO, USA).

### 2.3. Lentiviral Vector Preparation, Transduction of MEG-01 Cell Line and Cell Sorting

Lentiviral vectors were generated by transient co-transfection of HEK293T cells with a three-plasmid system: pHR’SINcppt-SE (carrying a GFP reporter gene), a VSV.G envelope plasmid and psPAX2 packaging plasmid, using LipoD293™ (SignaGen Laboratories, Frederick, MD, USA). For transduction, 1 mL of high-titer virus was added to 5 mL of MEG-01 cells in a T-25 flask. After 24 h, cells were centrifuged, and the medium was replaced with fresh RPMI 1640 containing antibiotics. Transduced cells were collected after 48 h, and GFP^+^ cells were sorted using a FACS Aria™ cytometer (BD Biosciences, Franklin Lakes, NJ, USA). These transduced cells will hereafter be referred to as MEG-01 GFP+.

### 2.4. MEG-01 Differentiation Protocol

MEG-01 or MEG-01 GFP+ cells were differentiated into megakaryocytes using 2 mM VPA. Control MEG-01 GFP+ cells were cultured at 10^5^ cells/mL in T-75 flasks with 15 mL EXOD-RPMI for 21 days, with partial medium replacement every 4 days to prevent oversaturation. VPA-treated MEG-01 or MEG-01 GFP+ cells were cultured under identical conditions with 2 mM VPA added, and the medium was refreshed every 4 days, adjusting the VPA final concentration to 2 mM. Cell populations were monitored throughout differentiation, and images were captured using an EVOS Fluorescence Microscope (Advance Microscopy Group (AMG), Life Technologies, Seattle, WA, USA).

### 2.5. Cell, Platelets and Extracellular Vesicle Isolation

MEG-01 or MEG-01 GFP+ cells and their derived populations were isolated by differential centrifugation. Cells were pelleted at 180× *g* for 10 min at 4 °C, and the supernatant was filtered through a 5 µm membrane. PLPs were collected at 1500× *g* for 15 min, MVs at 10,000× *g* for 40 min, and EXOs at 100,000× *g* for 120 min using a Beckman Coulter Optima XL ultracentrifuge equipped with a 70 Ti fixed-angle rotor.

In accordance with MISEV guidelines [20], the terms MVs and EXOs in this study designate operationally defined EV subtypes based on size, biogenesis, and isolation method. The vesicles isolated at 10,000× *g* for 40 min are classified as large EVs (lEVs), traditionally known as MVs, typically ranging from 100 to 1000 nm in diameter, enriched in canonical markers of megakaryocytic origin (e.g., CD41/CD42a), display externalized phosphatidylserine (PS) reflecting their plasma membrane budding origin and lack internal DNA. In contrast, vesicles collected at 100,000× *g* for 120 min fall in the 50–250 nm range, correspond to small EVs (sEVs, commonly called EXOs), and exhibited both megakaryocytic (CD41 and CD42a) and sEVs/EXO-associated (CD63) markers, consistent with their endosomal biogenesis.

MEG-01 GFP+ cells were employed for size characterization and visualization by imaging flow cytometry, while MEG-01 cells were used for proteomics assays to minimize potential artifacts introduced by lentiviral transduction and GFP overexpression [21].

Human platelet-rich plasma (PRP) was obtained from four healthy donors (n = 4). Individual samples were pooled to minimize inter-donor variability. The pooled blood was processed by centrifugation at 200× *g* for 20 min (no brake, acceleration set to 1), mixed with HEPES buffer containing 1 µM PGE1 to prevent activation, and pelleted at 800× *g* for 20 min (no brake, acceleration set to 1). Platelets were resuspended in Tyrode’s buffer, split into control and activated groups. Platelet activation was carried out at 37 °C, incubating with activation buffer (final concentration 1×) for 1 h. Platelet-derived EVs were isolated using the previously described differential centrifugation protocol.

### 2.6. Transmission Electron Microscopy (TEM)

TEM was employed to investigate the size and ultrastructure of VPA-differentiated MEG-01 GFP+ cells, their derived PLPs and EVs, as well as human platelets and their corresponding EVs. Samples were fixed in 2.5% glutaraldehyde and 2% paraformaldehyde (0.05 M cacodylate buffer, pH 7.4, 4 °C, 24 h), washed in 0.1 M cacodylate buffer, and post-fixed with 1% osmium tetroxide. They were sequentially stained with 1% tannic acid and 1% uranyl acetate, embedded in EMbed 812, and sectioned (50–70 nm) using a LEICA S ultramicrotome (Leica Microsystems, Wetztlar, Germany). Sections were counterstained with uranyl acetate and lead citrate before imaging. EVs were negatively stained with 1% uranyl acetate on carbon-coated grids (CF 300 Cu) for 5 min, stained for 1 min, and air-dried. Imaging was performed using a ZEISS Libra 120 plus TEM (120 kV, LaB_6_ filament) (Carl Zeiss Microscopy, Oberkochen, Germany).

### 2.7. Flow Cytometry Analysis

MEG-01 GFP+ cells and PLPs isolated from the VPA differentiation protocol were analyzed by flow cytometry. Cells were isolated and washed with PBS, centrifuged at 180× *g* for 10 min for cells and 1500× *g* for 15 min for PLPs, and incubated in the dark for 30 min with anti-CD41-PE (Invitrogen, ThemoFisher Scientific, Maltham, MA, USA) and anti-CD42a-APC (Miltenyi Biotec, Bergisch Gladbach, Germany) antibodies, diluted as recommended by the manufacturer. After washing, cells were resuspended in 100 µL PBS and analyzed with the FACS Canto II flow cytometer (BD Bioscience) using DIVA software (version 9.0, BD Bioscience). PLPs and platelet activation status were assessed using CD41-PE, CD42a-APC, and CD62P-BV421 (BD Bioscience) antibodies, with samples resuspended in PBS-EDTA (2 mM) post-washing, in the FACS Canto II flow cytometer (BD Bioscience).

### 2.8. ImageStream Analysis

MEG-01 GFP+ cells from the 21-day VPA protocol were isolated by centrifugation (180× *g* for 10 min). Cells were stained with anti-CD41-PE and anti-CD42a-APC, incubated for 15 min in the dark, fixed with 4% paraformaldehyde, permeabilized with 0.1% Triton X-100, and stained with Hoechst for 3 min. Platelets and PLPs were centrifuged at 800 or 1500× *g* for 15 min; stained with anti-CD41-PE, anti-CD42a-APC and the CD62P activation marker; incubated for 15 min; and resuspended in PBS-EDTA buffer (2 mM). MVs were obtained by centrifugation at 10,000× *g* for 40 min; stained with anti-CD41-PE, anti-CD42a-APC, 7AAD DNA marker, and Annexin V (Annexin V-CF Blue 7-AAD Kit (Abcam, Cambridge, UK)); and resuspended in Annexin buffer. EXOs were obtained by ultracentrifugation (100,000× *g* for 2 h); stained with anti-CD41-PE, anti-CD42a-APC, and anti-CD63-PE-Cy7 (BD Pharmingen, BD Bioscience); incubated for 40 min; and resuspended in PBS. Data were acquired using the ImageStream Mark II cytometer (Amnis, Seattle, WA, USA) and analyzed with IDEAS software (version 6.4, Amnis).

### 2.9. Nanoparticle Tracking Analysis

EV pellets (MVs at 10,000× *g*; EXO at 100,000× *g*) from MEG-01 GFP+ cells, platelets, and plasma were resuspended in 1 mL sterile PBS (pH 7.4). Measurements were performed using a Malvern NanoSight NS300 equipped with a 405 nm laser, sCMOS camera, and NTA software version 2.3 (Malvern Panalytical, Malvern, UK). Instrument performance was verified using 100 nm silica microspheres. Samples were diluted to the optimal detection range and injected at 50 µL/min at 25 °C. Five 10 s videos were recorded with different camera settings (CL = 8 and DT = 15 for MVs, CL = 10 and DT = 15 for EXOs), and size distribution and concentration were averaged using GraphPad Prism™ software (version 9.4.1 for Windows, La Joya, CA, USA). NTA measurements were performed on three biological replicates (independent EV preparations) with five technical replicates per sample, defined as five consecutive 60 s video recordings of the same aliquot to account for instrument variability.

### 2.10. Zeta Potential and Electrophoretic Mobility of Extracellular Vesicles (EVs)

Electrophoretic mobility (µe) and Zeta Potential (ZP) of the EV fractions were measured using a Malvern Zetasizer^®^ NanoZeta ZS (Malvern Panalytical). Fractions were resuspended in phosphate buffer (pH 7.4, 1.13 mM NaH_2_PO_4_) to prevent electrode damage and analyzed at 25 °C.

### 2.11. Protein Extraction, Digestion and Peptide Clean-Up

EVs from plasma, platelets, and MEG-01 cultures were lysed in 4.5% SDS and 0.1 M Tris-HCl (pH 8.5) with 5 mM tris(2-carboxyethyl)phosphine (TCEP) and 10 mM chloroacetamide (CAA). All samples were boiled at 95 °C for 10 min, sonicated for 10 min, and centrifugated to remove cellular debris. Protein concentration was determined using the Pierce BCA Protein Assay Kit (Thermo Fisher Scientific). Aliquots of 40 μg protein were digested using the single-pot, solid-phase (SP3) sample preparation method [22] with 100 µg/µL carboxylate-modified magnetic beads (GE Healthcare, Chicago, IL, USA) at a 10:1 bead:protein ratio (*w*/*w*). SDS was removed by washing twice with 80% (*v*/*v*) ethanol. Samples were acidified to pH 2–2.5 and desalted using an Oasis HLB 96-well µElution Plate (Waters, Milford, MA, USA) according to the manufacturer’s instructions. Peptides were resuspended in 0.5% formic acid and 2% acetonitrile, quantified by A280 measurements using NanoDrop (Thermo Fisher Scientific), and stored at −20 °C until Liquid Chromatography with tandem Mass Spectrometry (LC-MS/MS) analysis. All samples were processed in triplicate to ensure reproducibility and reliability of the results.

### 2.12. LC-MS/MS Analysis

Purified tryptic digests (200 ng peptide load) were separated using the predefined 15 SPD method (88 min gradient) on an Evosep One system (Evosep, Odense, Denmark) coupled online to a timsTOF Flex mass spectrometer (Bruker Daltonics, Germany). Peptides were delivered to a fused-silica emitter (20 µm ID; Bruker, Germany) mounted in a CaptiveSpray nanoelectrospray source (Bruker). The emitter was connected to a 15 cm × 150 µm reversed-phase analytical column packed with 1.9 µm C18 particles (PepSep Fifteen, Bruker). The column was maintained at 60 °C using a thermostated column oven (Bruker). Mobile phases consisted of water (A) and acetonitrile (B), both containing 0.1% formic acid (LC-MS grade, Fisher Scientific).

The ion mobility dimension was calibrated using three ions from the Agilent ESI-L Tuning Mix (*m*/*z*, 1/K_0_ values: 622.0289 Th, 0.9848 Vs cm^−2^; 922.0097 Th, 1.1895 Vs cm^−2^; 1221.9906 Th, 1.3820 Vs cm^−2^). Collision energy was linearly reduced from 59 eV at 1/K_0_ = 1.6 Vs cm^−2^ to 20 eV at 1/K_0_ = 0.6 Vs cm^−2^.

All samples were acquired using a DIA-PASEF method under the long-gradient configuration (*m*/*z* range: 400–1201 Th; 1/K_0_ range: 0.6–1.6 Vs cm^−2^; 16 × 25 Th diaPASEF windows).

### 2.13. Proteomics Data Analysis

Proteomic analyses were performed using three biological replicates (independent MEG-01 cultures and platelet samples) and five technical replicates (duplicate LC-MS/MS runs of each digested sample) to account for biological variability and instrument consistency. RAW MS data were processed using Spectronaut v18.1 (Biognosys AG, Zurich, Switzerland) for DIA analysis, searching a human proteome FASTA file (SwissProt, Geneva, Switzerland). All data analyses and visualizations were performed using the R language. Raw data was initially normalized using the standard cyclic loess normalization process [23] implemented in the limma package [24]. Several unsupervised hierarchical clustering analyses were performed and visualized as heatmaps using normalized and standardized protein abundance values (z-scores). All proteins commonly identified across the compared groups were included in the clustering, without any filtering. Clusters were generated using the complete-linkage algorithm based on Euclidean distances between samples [25]. Also, a dimensionality reduction study was applied using both a linear method (Principal Component Analysis, PCA) and a nonlinear method (t-distributed Stochastic Neighbor Embedding, t-SNE) [26] to analyze group clusterizations. Gene Ontology (GO) [27,28], Reactome [29], and Bioplanet [30] functional enrichments were performed using Genecodis, a bioinformatics tool that integrates diverse annotation sources [31]. Enrichment was conducted independently for the full set of proteins identified in each group. Additional functional analyses were generated through the use of IPA [32] and the Comparison analysis tool (QIAGEN Inc., Hilden, Germany, version 2024.1; https://www.qiagenbioinformatics.com/products/ingenuity-pathway-analysis, accessed on 31 October 2024). IPA was performed over differentially expressed proteins obtained between each natural product (Platelets, MVs and EXOs) and its corresponding MEG-01-derived populations. IPA provides activation z-scores indicating a predicted level of activation/inhibition in upstream regulators or canonical pathways.

### 2.14. Statistical Analysis

Data were analyzed using Microsoft^®^ Excel^®^ for Microsoft 365 MSO (64-bit, v2409), GraphPad Prism 9.4.1 and FlowJo V10 software (Treestar, Ashland, OR, USA). Values are expressed as mean ± standard deviation (SD). Differences between groups were assessed using unpaired two-tailed *t*-tests, Holm–Šídák’s multiple unpaired *t*-test, or one-way ANOVA. Furthermore, functional enrichment from GeneCodis provides statistical significance by hypergeometric and Wallenius tests corrected via Benjamini/Hochberg fold-discovery rate (FDR) [30]. An FDR-adjusted *p*-value < 0.01 was then considered for enrichment terms in GeneCodis. Also, relative enrichment scores are provided, measuring the proportion between the number of proteins for a specific term divided by the size of the input or the total number of proteins. The differentially expressed proteins included in the IPA were selected using a logFC > 1 and an adjusted *p*-value < 0.01, based on the false-discovery rate correction (Benjamini–Hochberg method) implemented in the *limma* R package (version 3.60.6) [24]. Finally, IPA for functional pathway enrichment and upstream regulator prediction was performed using the comparison analysis tool that combines *p*-value (Fisher’s exact test, *p*-value < 0.05) for enrichment and a bespoke activation z-score for directionality, allowing for direct, quantitative comparison of canonical pathway activity or upstream regulators across two datasets and visualization as a comparative heatmap (IPA, Qiagen Inc. version 2024.1).

## 3. Results

### 3.1. Fluorescence-Based Model for Tracking Cellular and Extracellular Vesicle Populations

To establish a reproducible in vitro model for studying megakaryocytic differentiation and EV production, we transduced the MEG-01 cells with the lentiviral vector pHR’SINcppt-SE, which constitutively expresses green fluorescent protein (GFP). Flow cytometry confirmed a transduction efficiency of 99.71 ± 0.12% (mean ± SD, n = 3) based on GFP expression compared to non-transduced controls. This fluorescence-based system, termed MEG-01 GFP+, enabled robust tracking of cellular populations (MEG-01 GFP+ cells, PLPs, MVs, and EXOs) throughout subsequent experiments.

### 3.2. Characterization of Megakaryocytic Differentiation in MEG-01 GFP+ Cells

**Figure 1:**
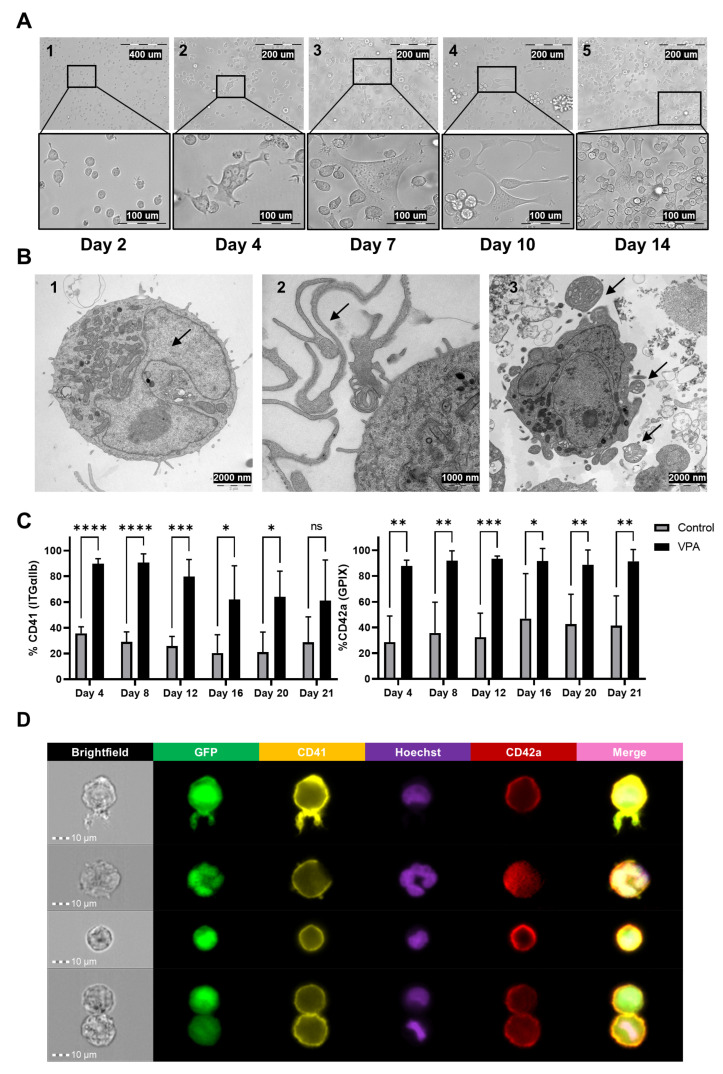
VPA-induced megakaryocytic differentiation of MEG-01 GFP+ cells. (**A**) Optical microscopy images of VPA-treated MEG-01 GFP+ cells captured on days 2, 4, 7, 10, and 14 at 20× (upper row) and 40× (lower row) magnification, illustrating morphological changes during differentiation. (**B**) Transmission electron microscopy images of VPA-treated MEG-01 GFP+ cells, highlighting ultrastructure changes during megakaryocytic differentiation. Arrows indicate key features. (**B1**) Presence of polylobulated nuclei in megakaryocytes; (**B2**) thick pseudopodia extending from the plasma membrane, contributing to proplatelet formation; (**B3**) release of proplatelets or nascent platelets via cytoplasmic membrane evaginations and pseudopodia. (**C**) Flow cytometry analysis of megakaryocytic surface markers CD41-PE and CD42a-APC in VPA-treated vs. control MEG-01 GFP+ cells, assessed every 4 days. Data represent the mean ± SD of quintuplicate experiments analyzed using Holm–Šídák’s multiple unpaired *t*-test: ns *p* > 0.05, * *p* ≤ 0.05, ** *p* ≤ 0.01, *** *p* ≤ 0.001, **** *p* ≤ 0.0001 vs. Control. (**D**) Representative ImageStream images of VPA-treated MEG-01 GFP+ cells, showing the localization and expression of GFP (cytoplasmic/nuclear, green), CD41-PE (plasma membrane, yellow), Hoechst (nuclear DNA, purple), and CD42a-APC (plasma membrane, red). The merged panel, excluding brightfield, integrates all channels, highlighting cellular organization during differentiation Black arrows indicate the key cellular structures identified in each panel: (**B1**) polylobulated nucleus, (**B2**) pseudopodia extension, (**B3**) proplatelets.

We employed a 21-day differentiation protocol using 2 mM VPA [19]. MEG-01 GFP+ cells subjected to treatment were replenished with fresh VPA-supplemented culture medium every 4 days, while control cells were maintained at appropriate confluence and nutrient levels without VPA exposure. Throughout the differentiation process, both culture images and TEM images were captured to evaluate the morphological and phenotypic changes in the MEG-01 GFP+ cell line (Figure 1A,B).

In the early stages of differentiation, most cells adhered to the flask surface, transitioning from suspension to adherence, which is necessary for differentiation initiation (Figure 1A, panel 1). By days 8–14, cells exhibited polylobed nuclei indicative of endoreplication, along with cytoplasmic expansion and the formation of membranous protrusions contributing to proplatelet development (Figure 1A, panels 2 and 3; Figure 1B, panel 1). These protrusions progressively elongate and branch, accumulating organelles and widening to form thick pseudopods from which proplatelets emerge (Figure 1A, panel 4; Figure 1B, panel 2). This process has been described as microtubule-driven in mature megakaryocytes [33], although microtubules are not directly visualized in our images. Proplatelet formation expands throughout the megakaryocyte cytoplasm until full conversion into proplatelet masses, which are released from the cell (Figure 1A, panel 5). Finally, the nucleus is expelled from the proplatelet mass, and individual platelets are released from proplatelet tips into the culture medium, generating PLPs.

In addition to morphological and phenotypic changes, flow cytometry analysis of megakaryocytic specific markers CD41 and CD42a revealed significantly higher expression in VPA-treated cells compared to controls throughout the differentiation protocol. On day 12, VPA-treated cells expressed CD41 at 74.55 ± 13.89% (vs. 26.83 ± 5.70% in controls, *p* < 0.001, n = 5) and CD42a at 90.81 ± 2.17% (vs. 38.03 ± 6.88%, *p* < 0.001, n = 5) (Figure 1C). Furthermore, imaging flow cytometry confirmed peripheral localization of CD41 and CD42a at the plasma membrane, ubiquitous GFP distribution, and polylobed nuclei stained with Hoechst, validating endoreplication (Figure 1D).

Collectively, these results demonstrate that VPA induces megakaryocytic differentiation with key morphological and immunophenotypic features.

### 3.3. Platelet-like Particles from VPA-Treated MEG-01 GFP+ Cells Mimic Activated Platelets

**Figure 2:**
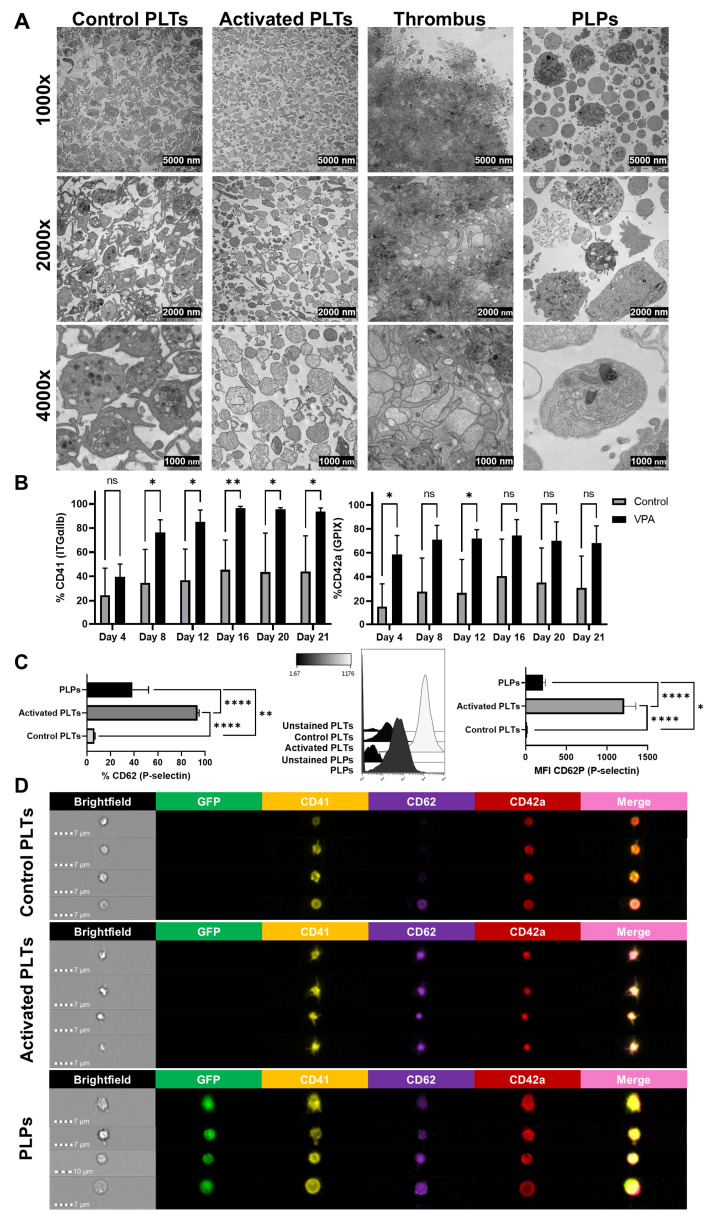
VPA-Driven Differentiation of MEG-01 GFP+ Cells Generates PLPs Resembling Activated Platelets. (**A**) Transmission electron microscopy images depicting ultrastructural features of platelets and PLPs across physiological and experimental conditions: (**A**) (1st row) Control platelets (Control PLTs), maintained in a resting state with PGE1, showing intact cytoplasmic granules and a discoid morphology; (2nd row) activated platelets (Activated PLTs), stimulated with an activation cocktail (collagen, ADP, and CaCl2), showing degranulation, membranous bodies, and cytoplasmic content release; (3rd row) thrombus-associated platelets, exhibiting isolated and Activated PLTs at the periphery and compact mass of Activated PLTs in the thrombus center; (4th row) PLPs from VPA-treated MEG-01 GFP+ cells, a heterogenous population resembling Activated PLTs (2nd row) but including MEG-01 fragments and necrotic debris. (**B**) Flow cytometry analysis of megakaryocytic differentiation markers (CD41-PE and CD42a-APC) in PLPs isolated by differential centrifugation from VPA-treated (2 mM) MEG-01 GFP+ cell supernatant. Differentiation analysis was assessed every four days. Data represent the mean ± SD (n = 5) analyzed by Holm–Šídák’s multiple unpaired *t*-test: ns *p* > 0.05, * *p* ≤ 0.05, ** *p* ≤ 0.01, **** *p* ≤ 0.0001 vs. Control. (**C**) Flow cytometry analysis of platelet activation marker CD62P-Pacific Blue (P-selectin) in Control PLTs, Activated PLTs, and PLPs. Both, Mean Fluorescence intensity (MFI) and percentage are shown. Data represent mean ± SD (n = 3) analyzed by one-way ANOVA: ns *p* > 0.05, * *p* ≤ 0.05, ** *p* ≤ 0.01, **** *p* ≤ 0.0001 vs. Control. (**D**) Representative ImageStream images of Control PLTs, Activated PLTs, and PLPs derived from VPA-treated MEG-01 GFP+ cells. Channels indicate localization and expression levels of GFP (cytoplasmic, green), CD41-PE (plasma membrane, yellow), CD62P-Pacific Blue (plasma membrane, purple), and CD42a-APC (plasma membrane, red). The merged panel, excluding brightfield, integrates all channels, highlighting phenotypic similarities and differences between platelets and PLPs.

VPA-induced PLPs were collected by centrifugation and compared to physiological platelets using TEM, flow cytometry and imaging flow cytometry. Figure 2A shows a comparative sequence of TEM images displaying the different physiological stages of platelets and PLPs. Control platelets (Control PLTs) were discoid and rounded (2–3 µm diameter), containing mitochondria and a well-defined canalicular system for granule secretion (e.g., alpha and dense granules). In contrast, activated platelets (Activated PLTs) were slightly smaller, with “empty” cytoplasm post-granule release, predominantly filled with protein and ribosomal material. Activated PLTs are tightly bound by fibrin-mediated membrane interactions within the thrombi, exhibiting a “vacant” cytoplasm. PLPs showed heterogeneity: most resembled activated PLTs in size (3–5 µm) and “empty” cytoplasm, while a smaller proportion resembled Control PLTs, with canalicular systems, granules, and mitochondria (4000× magnification). TEM also revealed fragmented MEG-01 GFP+ cells with nuclei pulled down during centrifugation (Figure 2A).

Flow cytometry assessed CD41 and CD42a expression in PLPs every 4 days, showing significantly elevated expression from VPA-treated cells compared to controls (CD41: 81.15 ± 21.73% vs. 38.18 ± 7.94%, *p* < 0.05, n = 5; CD42a: 69.11 ± 5.49% vs. 29.41 ± 8.66%, *p* < 0.05, n = 5), with stable levels by day 12 (Figure 2B). PLP activation was evaluated via CD62P (P-selectin) expression, present only on activated PLT membranes. PLPs exhibited higher CD62P levels (38.61 ± 13.57%) than Control PLTs (6.49 ± 0.53%, *p* < 0.01, n = 3) but lower than Activated PLTs (93.67 ± 1.25%, *p* < 0.0001, n = 3). The mean fluorescence intensity (MFI) of CD62P was significantly higher in PLPs than in Control PLTs (212 ± 30.01 vs. 19.56 ± 2.24; *p* < 0.05, n = 3), and, in turn, much higher in Activated PLTs (1187 ± 197.1; *p* < 0.0001, n = 3), showing a shift in the histogram plot (Figure 2C).

Imaging flow cytometry confirmed peripheral CD41 and CD42a localization at the plasma membrane in PLPs and platelets, with homogeneous CD62P expression in PLPs and activated PLTs but higher intensity in activated PLTs, while it was absent in Control PLTs (Figure 2D). Morphologically, Control PLTs appeared spherical, Activated PLTs present membrane extensions, and PLPs displayed heterogeneous patterns with larger size. GFP was present in PLPs, whereas it is absent in physiological platelets, allowing tracking due to inheritance from the VPA-treated MEG-01 GFP+ cell. Based on peak CD41 and CD42a expression, day 12 was selected to proceed with the study of the following vesicular populations.

### 3.4. Characterization of Microvesicles (MVs) from VPA-Treated MEG-01 GFP+ Cells

MVs are a subtype of EVs formed through the outward budding of the plasma membrane and typically range from 100 to 1000 nm in diameter. MVs reflect the molecular signature of their cell of origin and participate in physiological and pathological processes [34]. In order to further characterize the MVs produced by VPA-treated MEG-01 GFP+ cells (MVs of MEG-01), these EVs were isolated and subsequently compared to MVs from control platelets (MVs of Control PLTs), activated PLTs (MVs of Activated PLTs), and plasma (MVs of Plasma).

**Figure 3:**
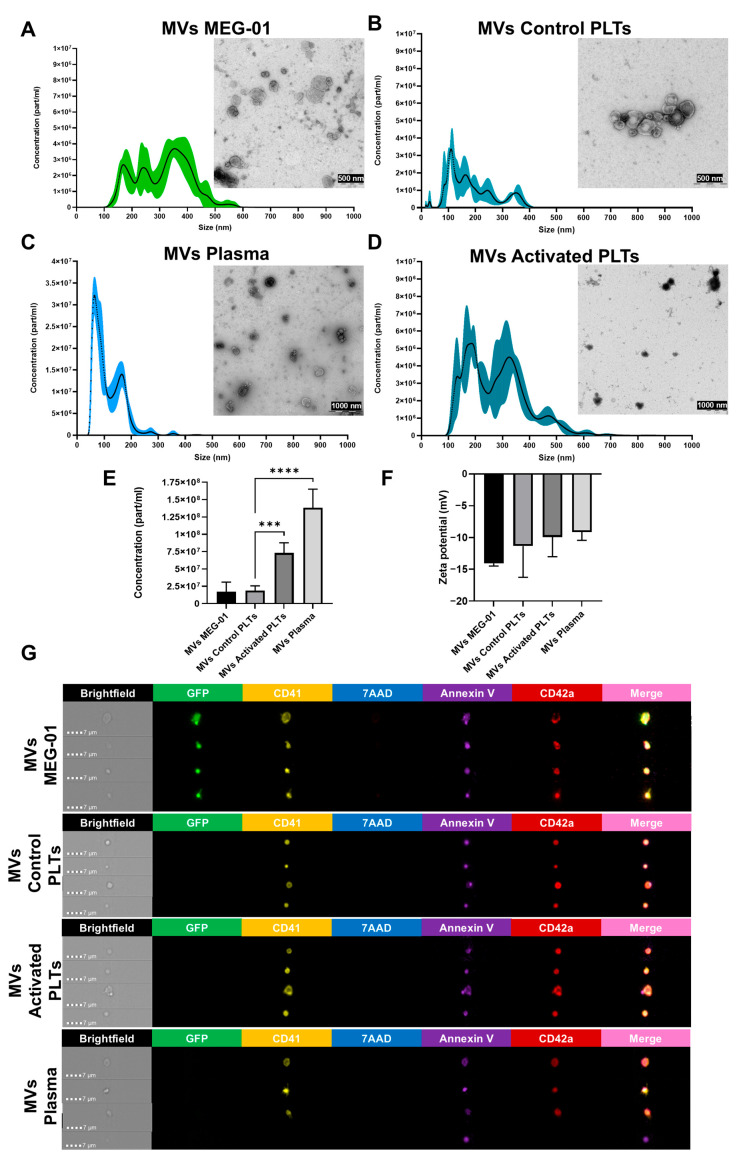
Phenotypic Characterization of Microvesicles (MVs) from VPA-treated MEG-01 GFP+ cells, Platelets and Plasma. (**A**–**D**) Size distributions (nm) of MVs derived from VPA-treated MEG-01 cells (**A**), Control (resting state) PLTs (**B**), Plasma (**C**), and Activated PLTs (**D**), measured by nanoparticle tracking analysis (NTA). Distributions represent the mean ± SD of five 10 s video analyses (n = 5). Representative transmission electron microscopy (TEM) images accompany each distribution, illustrating MVs size and morphology. (**E**) MV concentrations for each sample type (particles/mL of blood in the case of MVs of Plasma, Control and Activated PLTs, and particles/mL of media in the case of MVs of MEG-01), presented as mean ± SD from triplicate measurements (n = 3). Data represent mean ± SD (n = 3) analyzed by one-way ANOVA: ns *p* > 0.05, *** *p* ≤ 0.001, **** *p* ≤ 0.0001 vs. MVs of Control PLTs. (**F**) Zeta potential (mV) of MVs from each sample type, measured in triplicate and presented as mean ± SD (n = 3). (**G**) Representative ImageStream images of MVs from VPA-treated MEG-01 GFP+ cells, Control PLTs, Activated PLTs, and Plasma. Channels indicate localization and expression levels of GFP (cytoplasmic, GFP), CD41-PE (plasma membrane, yellow), 7AAD (DNA, pink), Annexin V-Pacific Blue (phosphatidylserine on plasma membrane, purple), and CD42a-APC (plasma membrane, red). The merged panel, excluding brightfield, integrates all channels, highlighting phenotypic profiles for each sample type and confirming the absence of apoptotic bodies.

Using NTA, a technique that measures particle size and concentration in suspension by analyzing their Brownian motion under laser illumination, we determined that MVs of MEG-01 ranged from 100 to 500 nm, with multimodal peaks at 170, 242, and 354 nm (Figure 3A). MVs of Control PLTs ranged from 100 to 400 nm with peaks at 110, 163, 244, and 351 nm (Figure 3B), while plasma-derived MVs (MVs of Plasma) were smaller (60–200 nm), with bimodal peaks at 64 and 165 nm (Figure 3C). Interestingly, MVs of Activated PLTs exhibited a similar size distribution to MVs of MEG-01, ranging from 100 to 500 nm with multimodal peaks at 186, 325, and 470 nm (Figure 3D). TEM confirmed vesicle sizes and morphological characteristics of different MVs populations (Figure 3A–D).

As previously indicated, NTA also allows precise quantification of particle concentration. MVs of Control PLTs had fewer particles/mL of blood than MVs of Activated PLTs (1.853 × 10^7^ ± 7.06 × 10^6^ particles/mL of blood vs. 7.29 × 10^7^ ± 1.47 × 10^7^ particles/mL of blood; *p* < 0.001; n = 3) and also a lower concentration than MVs of Plasma (1.383 × 10^8^ ± 2.687 × 10^7^ particles/mL of blood; *p* < 0.0001, n = 3), the latter being the MVs with the highest concentration. MVs of MEG-01 GFP+ exhibits concentrations similar to MVs of Control PLTs (1.7136 × 10^7^ ± 1.38 × 10^7^ particles/mL of media, n = 5) (Figure 3E). MVs were also assessed by their zeta potential, which measures the electrical potential between the vesicle membrane and the surrounding solution. Figure 3F shows that all MVs isolated from the different samples have negative potentials (−9.143 to −14.075 mV), ensuring biological origin and suspension stability and preventing aggregation due to electrostatic repulsion.

Phenotypic characterization by imaging flow cytometry assessed CD41, CD42a, Annexin V (indicating phosphatidylserine exposure), and 7-AAD (for DNA content). MVs of MEG-01 expressed GFP, Annexin V, and peripheral CD41 and CD42a, without 7AAD labeling, confirming their megakaryocytic origin and distinguishing them from apoptotic bodies (Figure 3G). MVs of Control PLTs and MVs of Activated PLTs lacked GFP and 7AAD but expressed Annexin V, CD41, and CD42a, while some MVs of Plasma lacked CD41 and CD42a, suggesting diverse cellular origins.

Together, these data indicate that MVs of MEG-01 closely mimics MVs of Activated PLT in size distribution and immunophenotype.

### 3.5. Characterization of Exosomes (EXOs) from VPA-Treated MEG-01 GFP+ Cells

**Figure 4:**
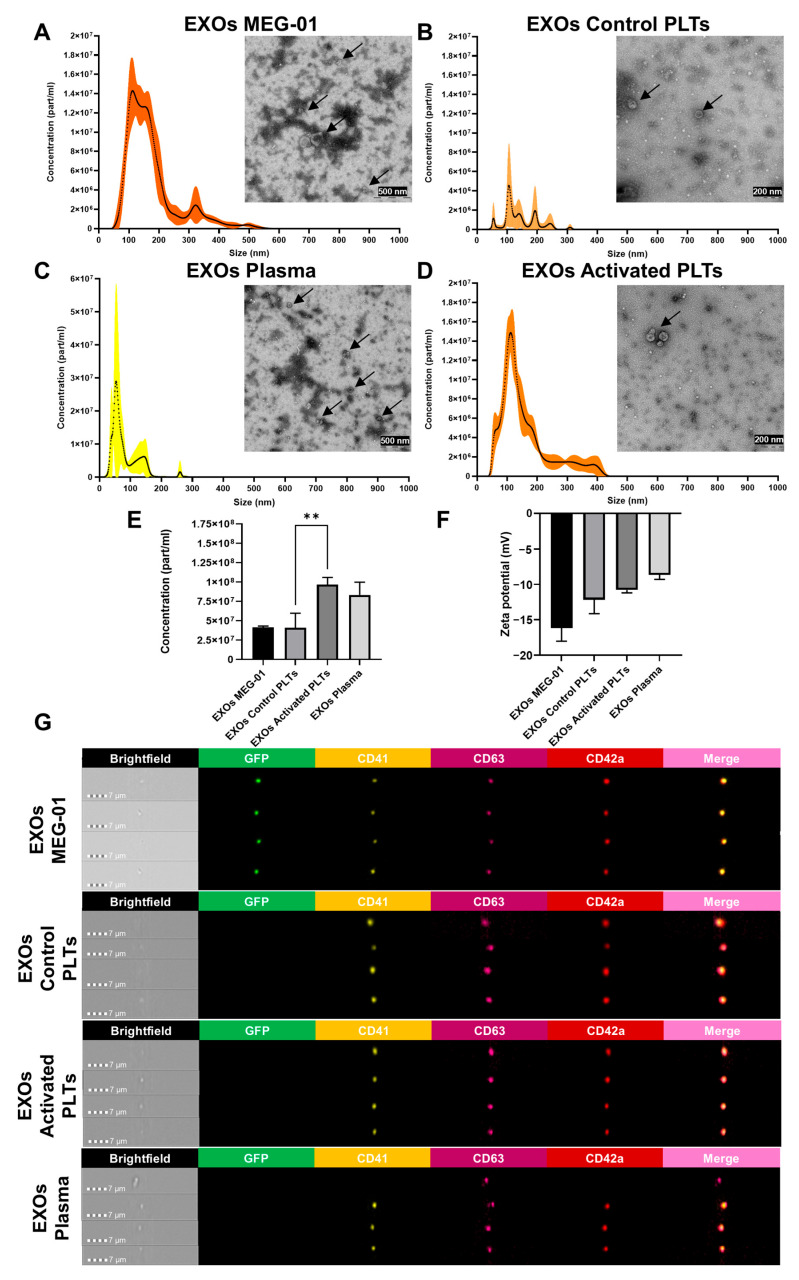
Phenotypic Characterization of Exosomes (EXOs) population from VPA-treated MEG-01 GFP+ cells. (**A**–**D**) Size distributions (nm) of EXOs from VPA-treated MEG-01 cells (**A**), Control or resting PLTs (**B**), Plasma (**C**), and Activated PLTs (**D**), measured by NTA. Distributions represent the average analysis of five 10 s videos, presented as mean ± SD (n = 5). Representative TEM images accompany each panel, illustrating EXO morphology and size (50–250 nm). (**E**) EXO concentrations for each sample (particles/mL of blood in the case of EXOs of Plasma, EXOs Control and EXOs of Activated PLTs, and particles/mL of media in the case of EXOs of MEG-01), presented as mean ± SD from triplicates (n = 3). Data represent mean ± SD (n = 3) analyzed by one-way ANOVA: ns *p* > 0.05, ** *p* ≤ 0.01 vs. EXOs of Control PLTs. (**F**) Zeta potential (mV) of the different EXO samples, measured in triplicate and presented as mean ± SD (n = 3). (**G**) Representative ImageStream images of EXOs generated by VPA-treated MEG-01 GFP+ cells, control platelets, activated platelets, and plasma. Channels show localization and expression levels of GFP (cytoplasmic, green), CD41-PE (plasma membrane, yellow), CD63-PECy7 (plasma membrane, pink), and CD42a-APC (plasma membrane, red). The merged panel, excluding the brightfield channel, combines all fluorescence channels to highlight the phenotypic profiles of each sample type. Black arrows highlight EXOs from the background.

EXOs are small EVs typically ranging from 50 to 250 nm in diameter, which originate from the endosomal pathway through the inward budding of multivesicular bodies (MVBs). Their biogenesis is characterized by the selective sorting of cargo via ESCRT (endosomal sorting complexes required for transport) machinery, distinguishing them from MVs [34]. For a comprehensive analysis, EXOs were isolated by ultracentrifugation at 100,000× *g* and subsequently compared among VPA-treated MEG-01 GFP+ cells (EXOs of MEG-01), control platelets (EXOs of Control PLTs), activated platelets (EXOs of Activated PLTs), and plasma (EXOs of Plasma). NTA and TEM analyses verified that EXOs ranged in size from 50 to 250 nm and exhibited characteristic morphology. EXOs of MEG-01 displayed a bimodal size distribution (peaks at 112 and 153 nm), resembling EXOs of Activated PLT (mode at 112 nm). EXOs of Control PLTs exhibit a multimodal distribution (peaks at 55, 107, 140, 193, and 244 nm), while EXOs of Plasma showed a bimodal distribution (54 and 144 nm) (Figure 4A–D).

Activated platelets produced a significantly higher concentration of EXOs of Activated PLTs (9.68 × 10^7^ ± 9.16 × 10^6^ particles/mL of blood) compared to EXOs control PLTs (4.083 × 10^7^ ± 1.89 × 10^7^ particles/mL of blood, *p* < 0.01, n = 3). EXOs of MEG-01 had a similar concentration to EXOs of Control PLTs (4.14 × 10^7^ ± 1.697 × 10^6^ particles/mL of media), whereas EXOs of Plasma had a similar concentration to EXOs of Activated PLTs (8.333 × 10^7^ ± 1.646 × 10^7^ particles/mL of blood) (Figure 4E). Zeta potential values ranged from −8.647 to −16.165 mV, indicating biological origin, colloidal stability, and lipid-rich composition in all samples (Figure 4F).

Phenotypic characterization was performed using imaging flow cytometry, assessing the expression of distinct markers (Figure 4G). In this case, CD63 was analyzed as a distinctive marker of MVB biogenesis. EXOs of MEG-01 expressed GFP, CD41/CD42a, and CD63, confirming their megakaryocytic and exosomal identity. EXOs of Control PLTs and EXOs of Activated PLTs similarly expressed CD41, CD42a, and CD63, verifying platelet origin. In contrast, some EXOs of Plasma lacked CD41 and CD42a, suggesting biosynthesis from non-platelets or megakaryocytic cell types (Figure 4G).

These results demonstrate that EXOs of MEG-01 share size distributions and CD41^+^/CD42a^+^/CD63^+^ profiles with platelet-derived EXOs.

### 3.6. Proteomic Analysis Reveals Distinct Signatures and Functional Specialization of MEG-01-Derived Vesicles

**Figure 5:**
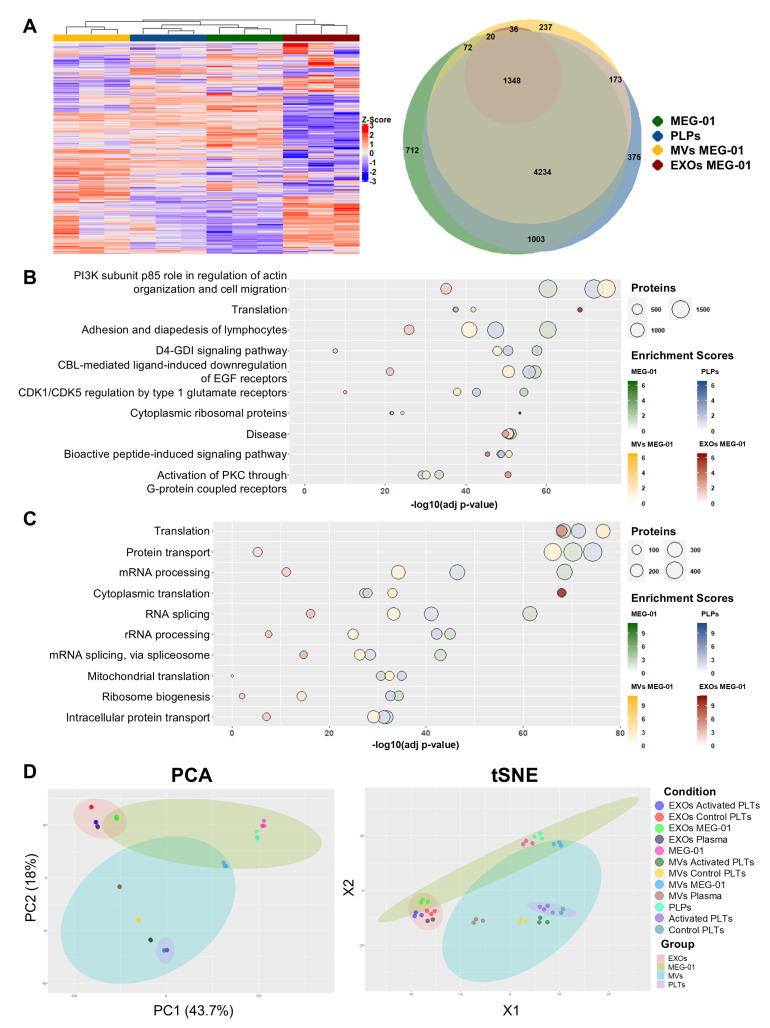
Proteomic and Functional Characterization of MEG-01 Cells and their Extracellular products. (**A**) Heatmap illustrating differentially expressed protein profiles across MEG-01 cells (green), platelet-like particles (PLPs) (blue), microvesicles (MVs) (yellow), and exosomes (EXOs) (brown). The accompanying Venn diagram highlights shared and unique protein subsets detected within each group. (**B**) BioPlanet pathways enrichment analysis, and (**C**) Gene Ontology (GO) Biological Process analysis, each presenting the top 10 most significantly enriched biological processes or pathways ranked by adjusted *p*-value. The diameter of each bubble denotes the number of proteins associated with a given pathway or process, while bubble color intensity reflects the enrichment score for each individual sample type: MEG-01 cells (green), PLPs (blue), MVs (yellow), and EXOs (brown). (**D**) Principal Component Analysis (PCA) and t-distributed Stochastic Neighbor Embedding (t-SNE) maps segregating MEG-01 cells, PLPs, MVs, and EXOs, and their natural human platelet-derived counterparts: Platelets (PLTs), as well as associated MVs and EXOs from Control or Activated PLTs. Each sample replicate is mapped individually, maintaining a group-specific color scheme. MEG-01-derived products are grouped within a green area, platelets in purple, MVs in light blue, and EXOs in orange, illustrating distinct clustering and phenotypic boundaries between these populations.

To comprehensively characterize the proteomic landscape of VPA-treated MEG-01 cells (MEG-01) and their derivatives (PLPs, MVs of MEG-01 and EXOs of MEG-01), we analyzed protein abundance profiles across multiple sample types using quantitative LC-MS/MS. Unsupervised hierarchical clustering analysis revealed clear compartment-specific expression patterns (Figure 5A). The heatmap demonstrated coherent clustering within sample classes, with expression gradients indicative of distinct proteomic signatures for each fraction type. Complementary Venn diagrams illustrated the intersection of identified proteins across the four main fractions, revealing substantial overlaps in core proteomes while highlighting unique protein subsets specific to each compartment. These findings suggest both shared biological functions and specialized cargo-sorting mechanisms operate during PLP/EV biogenesis.

To elucidate the functional significance of these proteomic profiles, we performed pathway enrichment analysis using GeneCodis. The top-10 most significant terms with FDR-adjusted *p*-value < 0.01 were considered. Bioplanet pathway analysis revealed strong enrichment of specific signaling modules within vesicle fractions (Figure 5B). Pathways related to actin cytoskeleton organization and cell migration (PI3K subunit p85’s role in regulation of actin organization and cell migration), Rho family regulation (D4-GDI signaling pathway), and receptor-mediated signaling (CBL-mediated ligand-induced downregulation of EGF receptors) showed particularly higher significance in MEG-01 cells, PLPs and MVs of MEG-01 compared to EXOs of MEG-01. In contrast, protein biosynthesis pathways were more prominent in EXOs of MEG-01, showing the highest enrichment scores in the Translation and Cytoplasmic ribosomal protein categories.

Gene Ontology (GO) Biological Process analysis demonstrated that all fractions were strongly enriched in pathways governing RNA and protein biosynthesis (Figure 5C). Notably, enrichment scores for mRNA biosynthetic pathways (mRNA processing, RNA splicing and mRNA splicing via spliceosome) were highest in parent MEG-01 cells and progressively declined in derived vesicles, suggesting dilution or functional specialization of this machinery during vesicle formation. Ribosome biosynthesis pathways (Ribosome biogenesis and rRNA processing) displayed elevated enrichment in MEG-01 cells and PLPs, whereas protein intracellular trafficking pathways (Protein transport and Intracellular protein transport) and Mitochondrial translation showed reduced significance in EXOs of MEG-01. Reactome analysis (Supplementary Figure S1A) corroborated these findings, highlighting dominant overrepresentation of RNA metabolism and translation pathways across all fractions.

Collectively, this functional profiling indicates a transition from a primary biosynthetic role within MEG-01 cells to specialized functions in intercellular communication and cell signaling within derived EVs.

To obtain a global perspective on the proteomic landscape of MEG-01 derivatives relative to their natural counterparts, we performed unsupervised dimensionality reduction techniques, including principal component analysis (PCA) and t-distributed stochastic neighbor embedding (tSNE) on the complete dataset (Figure 5D). PCA revealed clear separation of experimental groups, with the first principal component accounting for 43.7% of the variance, clearly separating MEG-01 cells, platelets, MVs and EXOs. The second principal component (18%) allowed further sub-clustering, confirming high intragroup consistency and distinct molecular identities across groups. Notably, PLPs (cyan color) segregated more closely with MEG-01 cells (pink color) than with native platelets, regardless of activation status. Independent t-SNE analysis confirmed these PCA results, producing tight, well-separated clusters corresponding to each sample group and experimental condition.

GO Cellular Component analysis revealed significant enrichment of the Cytoplasm, Nucleoplasm and Mitochondrion terms in PLPs, mirroring the profile observed in parental MEG-01 cells. In contrast, MVs of MEG-01 and EXOs of MEG-01 exhibited notable enrichment in the Extracellular exosome and Blood microparticle terms, respectively (Supplementary Figure S1B). These findings suggest that PLPs retain substantial intracellular components, which may arise from arrested maturation toward a terminally differentiated platelet phenotype or from contamination with MEG-01-derived cellular debris during the isolation protocol.

### 3.7. Functional Proteomic Profiling Distinguishes PLPs from Native Platelets and MEG-01 Cells

**Figure 6:**
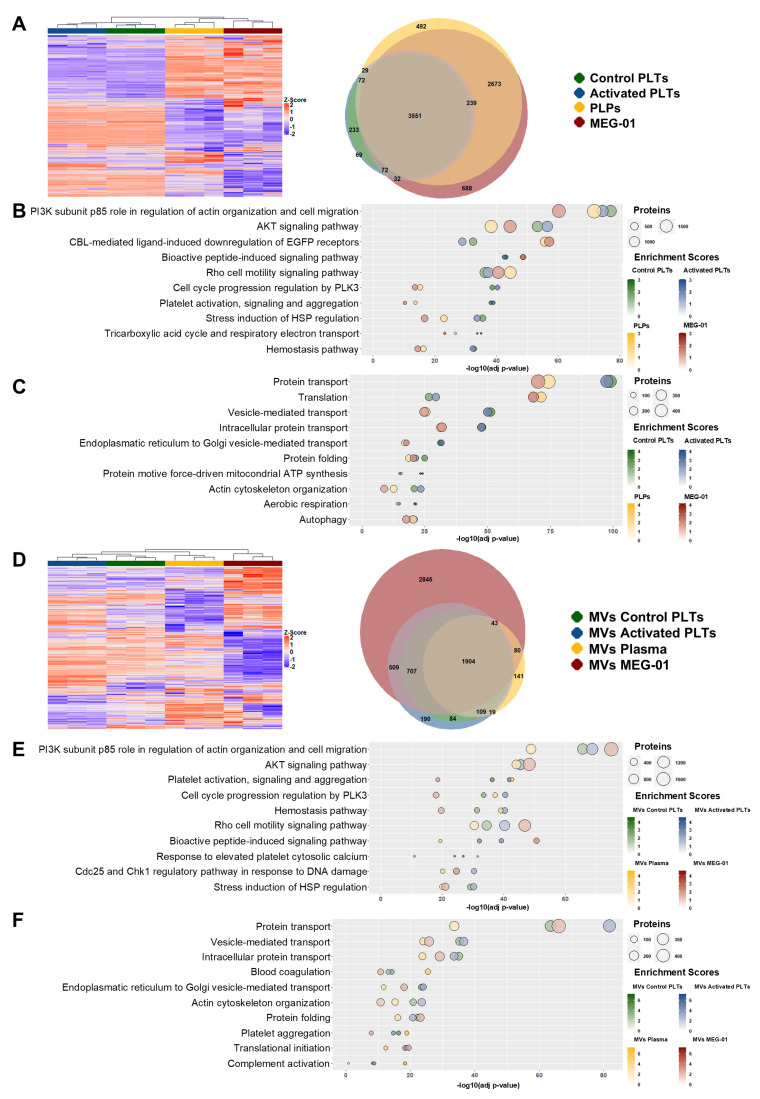
Comparative Proteomic and Functional Analysis of Human Platelets, MEG-01 Cells, and their derived populations. (**A**) Heatmap illustrating differential protein expression across Control Platelets (green), Activated Platelets (blue), PLPs (yellow), and MEG-01 cells (brown). The accompanying Venn diagram shows shared and unique protein subsets, revealing both overlapping and distinct molecular signatures. (**B**) BioPlanet pathway enrichment analysis showing the top 10 most significantly enriched biological pathways, ranked by adjusted *p*-value, for each sample type. Bubble size indicates the number of proteins mapped to each pathway, and color intensity reflects the enrichment score. (**C**) Gene Ontology (GO) Biological Process enrichment analysis presenting the top 10 most enriched biological processes for each group. Bubble diameter represents the number of associated proteins, and color intensity indicates the enrichment score per group. (**D**) Heatmap and Venn diagram depicting differential protein expressions in MVs from Control Platelets (green), Activated Platelets (blue), Plasma (yellow), and MEG-01 cells (brown). (**E**) BioPlanet pathway enrichment analysis of MVs from Control Platelets, Activated Platelets, Plasma, and MEG-01 cells, showing the top 10 most enriched pathways. Bubble size reflects the number of proteins, and color intensity indicates the enrichment score. (**F**) GO Biological Process enrichment analysis of MVs, presenting the top 10 most enriched processes. Bubble diameter corresponds to protein count, and color intensity reflects the enrichment score for each group.

Hierarchical clustering of protein abundance z-scores demonstrated clear segregation of native platelets (control and activated), PLPs and MEG-01 cells into distinct groups (Figure 6A). Venn diagram analysis revealed a substantial core proteome of 3551 proteins shared across all samples, alongside fraction-specific subsets comprising 492 proteins unique to PLPs and 688 unique to MEG-01 cells, indicating selective protein sorting during PLP biogenesis from MEG-01 precursors. Interestingly, platelet activation resulted in the detection of 69 proteins and the loss of 239 proteins compared to resting platelets (Figure 6A).

Functional enrichment analysis across multiple databases revealed convergent proteomic signatures. BioPlanet pathway analysis identified significant overrepresentation of cytoskeletal and signaling modules, together with platelet-specific programs and metabolic pathways (Figure 6B). GO Biological Process analysis corroborated these findings, demonstrating strong enrichment in protein and vesicle trafficking, protein biosynthesis and cellular respiration (Figure 6C). Reactome pathway analysis further confirmed dominant enrichment in protein trafficking, immune-related programs and platelet-specific pathways (Supplementary Figure S2A). These results indicate that PLP proteomes appear closer to MEG-01 cells than platelets, likely influenced by residual debris or incomplete maturation.

GO Cellular Component analysis localized the identified proteins predominantly to Extracellular exosome and Cytoplasm compartments, with substantial contributions of mitochondrial fraction (Mitochondrion) (Supplementary Figure S2B). Remarkably, nuclear proteins (Nucleoplasm) remained present in PLPs, suggesting co-purification of cellular debris containing nuclear components from MEG-01 cells during the isolation process.

### 3.8. Comparative Proteomics Identifies Conserved Hemostatic Programs in Platelet, Plasma and MEG-01-Derived MVs

Proteomic analysis of MVs of Control PLTs, MVs of Activated PLTs, MVs of Plasma, and MVs of MEG-01 demonstrated robust sample discrimination through hierarchical clustering, while Venn analysis identified both shared and fraction-specific protein signatures (Figure 6D). A substantial core proteome of 1904 proteins was present across all MV sources, indicative of common vesicular biogenesis mechanisms. However, significant differences emerged: MVs of MEG-01 contained 2846 unique proteins, reflecting their distinct cellular origin. MVs of Plasma exhibited 221 differentially expressed proteins compared to platelet MVs, with 141 being unique to this fraction. Interestingly, MVs of Activated PLTs showed dramatic cargo enrichment, gaining 761 proteins upon activation, 190 of which were exclusive to this activated state.

Functional enrichment analysis revealed that MV proteomes were dominated by hemostatic and signaling pathways across all sources. BioPlanet pathway analysis emphasized cytoskeletal remodeling, platelet activation programs, and stress/checkpoint responses (Figure 6E). GO Biological Process terms concentrated on protein trafficking, protein biosynthesis, and hemostatic functions (Figure 6F). Reactome analysis reinforced these patterns through enrichment in trafficking, hemostasis, and immune surveillance pathways (Supplementary Figure S3A).

GO Cellular Component enrichment localized MV proteomes predominantly to Extracellular exosome and Cytoplasm compartments (Supplementary Figure S3B). Fraction-specific differences emerged in organellar protein content: mitochondrial proteins showed reduced enrichment in MVs of Control PLTs, MVs of Activated PLTs and MVs of Plasma relative to MVs of MEG-01, while nuclear proteins were uniquely present in MVs of MEG-01, which may indicate isolation of intracellular debris containing nuclear fragments from the parent cell line.

In summary, these results highlight the central role of conserved hemostatic and communication pathways across all MVs while also revealing distinct molecular signatures that reflect their diverse cellular origins.

### 3.9. Exosome Proteomic Characterization Reveals Source-Specific Signatures and Conserved Hemostatic Functions

**Figure 7:**
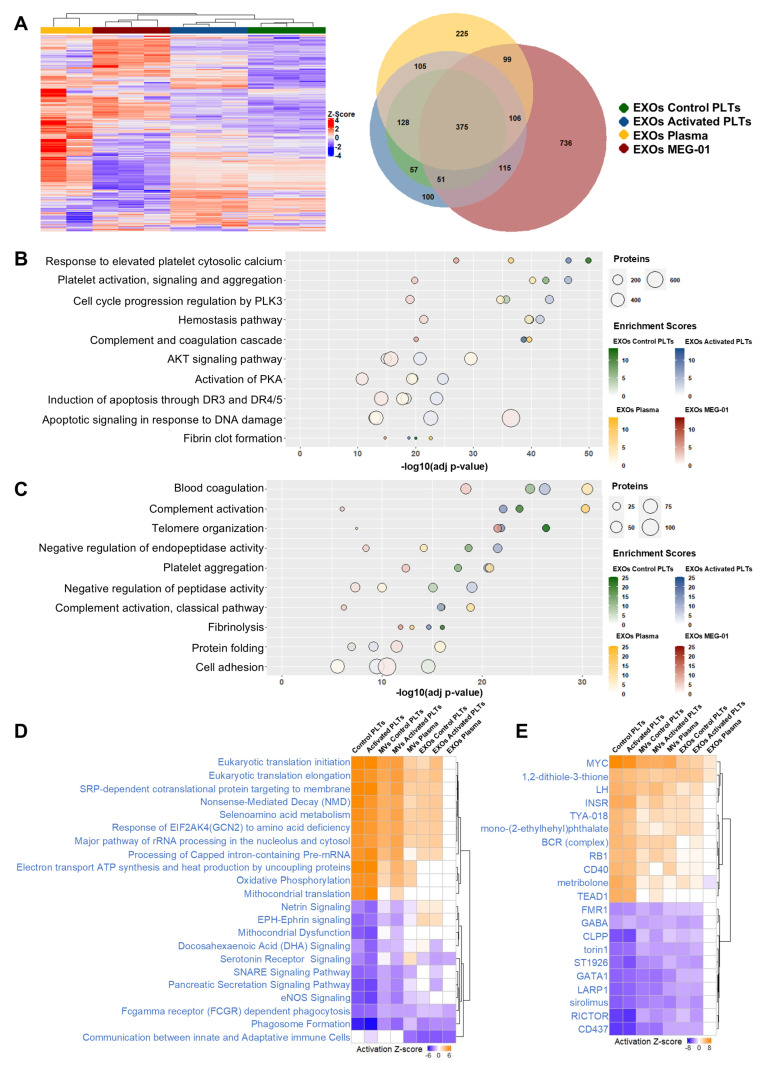
Comparative Functional and Upstream Regulator Analysis of Human Platelets, Plasma, MEG-01 Cells, and their extracellular vesicles. (**A**) Heatmap illustrating differential protein expression profiles of key biological functions across EXOs purified from Control Platelets (green), Activated Platelets (blue), Plasma (yellow), and MEG-01-cells (brown). The accompanying Venn diagram highlights shared and unique protein subsets, revealing overlapping and distinct molecular signatures. (**B**) BioPlanet pathway enrichment analysis showing the top 10 most significantly enriched biological pathways, ranked by adjusted *p*-value, for EXOs of Control PLTs, EXOs of Activated PLTs, EXOs of Plasma and EXOs of MEG-01. Bubble size indicates the number of proteins mapped to each pathway, and color intensity reflects the enrichment score. (**C**) Gene Ontology (GO) Biological Process enrichment analysis presenting the top 10 most enriched biological processes for each sample type. Bubble diameter represents the number of associated proteins, and color intensity indicates the enrichment score for EXOs of Control Platelets, EXOs of Activated Platelet, EXOs of Plasma, and EXOs of MEG-01. (**D**) Ingenuity Pathway Analysis (IPA) comparing key biological functions inferred from relative protein expression in natural products (Platelets, MVs and EXOs) and their MEG-01-derived counterparts (PLPs, MVs of MEG-01 and EXOs of MEG-01). This panel highlights the 10 top most differentially up- or down-regulated functions distinguishing native and engineered populations. (**E**) IPA-based prediction of the most significantly relevant upstream regulatory mediators driving proteomic differences between natural and MEG-01-derived populations.

EXO proteomes displayed population-specific signatures alongside a conserved core of 375 proteins (Figure 7A). Hierarchical clustering via heatmap visualization demonstrated robust segregation of EXOs samples by cellular origin. Venn diagram analysis identified 100 proteins unique to EXOs of Activated PLTs, 225 to EXOs of Plasma and 736 to EXOs of MEG-01. These patterns reflect both conserved vesicle biogenesis machinery and source-dependent cargo selection characteristic of the endosomal pathway.

BioPlanet enrichment analysis confirmed that EXOs exhibited proteomic enrichment for hemostatic and immune pathways, although functional activity was not directly assessed. A strong representation of platelet activation programs, coagulation cascades, cell signaling modules, cell cycle regulation, and apoptotic responses was obtained (Figure 7B). GO Biological Process enrichment corroborated these hemostatic signatures, proteolytic regulation, and general cellular processes (Figure 7C).

Reactome analysis reinforced immune and platelet-specific functions, featuring enriched categories, including Hemostasis, Platelet activation, signaling and aggregation, Response to elevated platelet cytosolic Ca^2+^, Platelet degranulation, Neutrophil degranulation, and Complement cascade, consistent with platelet-origin EV biology (Supplementary Figure S4A).

Subcellular localization analysis revealed a defining characteristic of EXOs: proteins localized predominantly to Extracellular exosome and Blood microparticle compartments, with markedly reduced mitochondrial and nucleoplasmic content compared to PLPs and MVs of MEG-01 (Supplementary Figure S4B). This compositional refinement reflects the selective ESCRT-dependent sorting mechanisms that distinguish EXOs biogenesis through MVBs from the direct membrane budding processes generating MVs and the differentiation pathway producing PLPs.

### 3.10. Differential Pathway Analysis Reveals Striking Functional Convergence Between EXOs of Plasma and EXOs of MEG-01

To identify molecular networks and regulatory mechanisms distinguishing native platelets and their EVs from MEG-01-derived counterparts, differential proteomic analysis was performed using Ingenuity Pathway Analysis (IPA). Native platelets and EVs from platelets and plasma were normalized to their respective MEG-01-derived fractions (PLPs, MVs of MEG-01 and EXOs of MEG-01), allowing direct assessment of functional similarity. In the resulting heatmaps, orange indicates pathways or upstream regulators more highly activated in MEG-01 fractions, blue indicates reduced activation, and white represents minimal differential activation, signifying functional convergence between populations (Figure 7D,E).

Native platelets showed pronounced downregulation of biosynthetic pathways, including mRNA maturation (Processing of Capped intron-containing Pre-mRNA), ribosome biogenesis (Major pathway of rRNA processing in the nucleolus and cytosol), and translational process (Eukaryotic translation initiation, Eukaryotic translation elongation, Mitochondrial translation, Nonsense-Mediated Decay and SRP-dependent cotranslational protein targeting to membrane), alongside mitochondrial bioenergetics (Oxidative Phosphorylation). Conversely, platelets exhibited increased activation of multiple signaling networks (Netrin Signaling, EPH-Ephrin signaling, Docosahexaenoic Acid (DHA) Signaling, Serotonin Receptor Signaling, SNARE Signaling Pathway and eNOS Signaling) and Phagosome Formation compared to PLPs (Figure 7D).

Platelet-derived MVs displayed a nearly identical functional pattern, with downregulation of biosynthetic and bioenergetic pathways and upregulation of some signaling pathways (EPH-Ephrin signaling, Docosahexaenoic Acid (DHA) Signaling, Serotonin Receptor Signaling, SNARE Signaling Pathway and eNOS Signaling) and Phagosome Formation (Figure 7D). MVs of Plasma exhibited the same downregulation profile but with attenuated z-score intensities while maintaining only the SNARE Signaling Pathway. Interestingly, MVs of Plasma uniquely displayed communication between innate and adaptative immune Cells, a function absent in platelet-derived MVs and MVs of MEG-01 (Figure 7D).

In striking contrast, EXOs of Plasma showed minimal differential pathway activation compared to EXOs of MEG-01, evidenced by predominantly white coloring throughout the heatmap (Figure 7D). This remarkable convergence suggests that EXOs of Plasma are significantly enriched in megakaryocyte-derived vesicles. Platelet-derived EXOs, however, retained distinct downregulation of biosynthetic pathways—mRNA maturation, ribosome biogenesis and translational regulation—differentiating them from both EXOs of MEG-01 and EXOs of Plasma (Figure 7D).

Upstream regulator analysis with IPA revealed contrasting transcriptional programs between native platelets and MEG-01-derived PLPs (Figure 7E). Proliferation-associated transcription factors—MYC, RB1 and TEAD1 (a direct activator of MYC in the YAP/TAZ-TEAD pathway) [35]—were predicted to be more highly activated in PLPs. In contrast, GATA1, the master regulator of terminal megakaryocyte differentiation, showed elevated activation in native platelets. These findings indicate that despite VPA treatment, MEG-01 cells maintain proliferative transcriptional programs and fail to achieve complete terminal megakaryocytic differentiation, which would be characterized by GATA1 dominance and suppression of proliferative drivers like MYC.

Additional upstream regulators distinguishing native platelets from MEG-01-derived fractions included receptor-mediated signaling pathways (BCR complex, CD40, INSR, and LH) and pharmacological modulators (1,2-dithiole-3-thione, TYA-018, mono-(2-ethylhexyl)phthalate, and metribolone) (Figure 7E). In contrast, native platelets displayed activation of mTOR pathway components, RICTOR (mTORC2), LARP1 (translational regulator downstream of mTORC1), and also mTOR inhibitors sirolimus/Rapamycin and torin 1 are suggested to be upregulated. In addition, FMR1, an RNA-binding protein regulating mRNA splicing, stability, and localized translation, is upregulated in native platelets. FMR1’s function parallels the selective mRNA trafficking and compartmentalized translation in neurons [35], a similar process that occurs during proplatelet formation in mature megakaryocytes, suggesting native platelets retain sophisticated post-transcriptional control machinery diminished in MEG-01-derived PLPs.

Importantly, EXOs of Plasma showed minimal upstream regulator differences from EXOs of MEG-01, reinforcing their functional convergence observed in pathway analysis (Figure 7E). This remarkable similarity contrasts sharply with the distinct proteomic identities maintained by native platelets and platelet-derived EVs relative to MEG-01-derived vesicles. Consistent with previous reports by Flaumenhaft et al. [36], our proteomic analysis confirms that EXOs of MEG-01 exhibit an expression profile characteristic of megakaryocyte-derived EVs. Specifically, these EXOs express CD41 and CD42b but lack the activation marker CD62P while retaining full-length filamin A (Supplementary Figure S4C). This profile distinguishes them from platelet-derived EXOs, which display CD62P and cleaved filamin A.

Our findings indicate that EXOs of MEG-01 exhibit greater similarity to megakaryocyte-lineage vesicles, thereby supporting their potential utility as surrogate models in experimental studies.

## 4. Discussion

This study provides a comprehensive proteomic assessment of MEG-01-derived EVs as surrogate models for selected aspects of platelet EV biology. Through integrative analysis combining morphological characterization, quantitative proteomics, and pathway enrichment, we show that VPA-treated MEG-01 cells generate PLPs, MVs, and EXOs that reproduce several structural and functional features of platelet-derived vesicles, while differences remain in terms of activation state and maturation.

Our findings indicate that VPA-induced PLPs exhibit an activated platelet phenotype with elevated CD62P expression, degranulated morphology, and “empty” cytoplasmic appearance characteristic of post-secretory platelets. Despite these genotypic and phenotypic similarities, two major challenges prevent complete substitution of native platelets in experimental settings. First, the yield of PLPs obtained in vitro remains far below physiological platelet concentrations found in blood, though this limitation could potentially be addressed through bioreactor systems that enhance MEG-01 differentiation and facilitate PLP release and isolation. Second, PLPs predominantly resemble activated platelets with empty cytoplasm post-degranulation, suggesting that PLPs undergo constitutive activation during their generation, leading to premature release of granular contents. This activation-like state limits their utility for studying quiescent platelet biology or early activation events but positions them as valuable models for investigating activated platelet functions relevant to thrombosis and cancer-associated platelet activation.

Optimization of PLP purification protocols represents an immediate opportunity to enhance model fidelity by minimizing cellular debris contamination. Our current differential centrifugation protocol effectively separates PLPs from parent cells but co-purifies nuclear fragments and cellular debris, as evidenced by TEM and the persistence of nucleoplasmic proteins in proteomic analysis. Several alternative purification strategies may be evaluated; however, each method presents inherent compromises between PLP yield, sample purity, processing complexity and scalability for experimental applications.

First, density gradient ultracentrifugation using discontinuous iodixanol gradients (40–60% gradients) could exploit density differences between intact PLPs (specific gravity ~1.03–1.04 g/mL, similar to native platelets) and cellular debris to achieve superior separation [37]. Second, immunoaffinity purification leveraging positive selection for CD41/CD61-expressing particles through magnetic bead or affinity column separation could provide highly specific PLP isolation while excluding nucleated cells and debris [38]. Comparative proteomic validation of these methods using nucleoplasmic marker quantification, such us histones or nuclear pore proteins, would establish standardized protocols for research-grade PLP production.

Beyond purification optimization, improving terminal differentiation could generate PLPs with more physiologically authentic transcriptional profiles. Our data demonstrate that despite VPA-induced morphological maturation, MEG-01 cells fail to achieve GATA1-dominant, MYC-suppressed transcription characteristic of terminal megakaryopoiesis. Amplifying GATA1 function represents the most direct approach. While combinatorial treatment with VPA and thrombopoietin receptor agonists, such as romiplostim or eltrombopag, could synergistically activate GATA1-dependent transcription through JAK2/STAT5 pathways, genetic strategies offer more precise control [39,40]. Our previous work demonstrated that SCL/TAL1-driven transcriptional networks strongly promote megakaryocytic specification from human embryonic stem cells (hESCs), where TAL1 cooperates with GATA1, LMO2, and related cofactors to reinforce lineage commitment [14]. Building on this concept, Moreau et al. developed a megakaryocytic differentiation protocol from human-induced pluripotent stem cells (hiPSCs) using lentiviral transduction of three key transcription factors, FLI1, TAL1, and GATA1, which act synergistically to govern megakaryocytic maturation [41]. Following these insights, we utilized the GATA1–FLI1–TAL1 lentiviral system to recapitulate macrothrombocytopenia in an hiPSC model of Bernard-Soulier Syndrome Type C. This approach successfully recapitulated the macrothrombocytopenia phenotype in vitro, which we subsequently restored through GP9-based gene therapy [42]. Collectively, these forward programming strategies enable highly efficient megakaryocyte differentiation in hiPSCs.

Suppressing c-MYC activity represents a complementary regulatory mechanism during megakaryopoiesis. Bourquin et al. demonstrated that in acute megakaryoblastic leukemia (AMKL) patients, altered GATA1 function leads to a failure to repress proliferation-associated genes such as c-MYC, contributing to disease pathology [43]. Takayama et al. (2010) further showed, using a doxycycline-inducible system, that transient activation of c-MYC is essential for megakaryocyte differentiation from hiPSCs, whereas sustained c-MYC expression beyond the megakaryocyte progenitor stage impairs maturation and reduces platelet release [44]. Pharmacologically, c-MYC inhibition offers practical approaches without requiring genetic manipulation. Small molecule c-MYC inhibitors, including 10058-F4 and OMO-103, disrupt MYC-MAX heterodimerization [45], while BET bromodomain inhibitors, such as I-BET-762, block MYC transcriptional activity through chromatin remodeling [46]. These findings highlight the possibility of precise temporal control of c-MYC suppression during the critical phase of megakaryocyte maturation, shifting the differentiation balance toward terminal maturation driven by GATA1 dominance.

Beyond VPA, alternative HDAC inhibitors may offer improved terminal differentiation. We and other groups have described that the effectiveness of HDAC inhibitors in promoting megakaryopoiesis and thrombopoiesis varies considerably across cellular models [14]. Comparative studies evaluating these compounds, individually or in combination, would establish optimized differentiation protocols yielding PLPs with native-like transcriptional profiles characterized by elevated GATA1, suppressed MYC, and enhanced functional capacity. However, not all HDAC inhibitors are effective for promoting megakaryopoiesis and thrombopoiesis, as their impact varies depending on the specific HDAC target and cellular context. Our upstream regulator analysis identified TYA-018, a selective HDAC6 inhibitor [47], suggesting that HDAC6 activity represents a conserved regulatory node distinguishing native platelets from MEG-01-derived populations. Importantly, in humans, HDAC6-selective inhibition preserves nuclear HDAC1/2/3 activity while inducing hyperacetylation of cortactin (CTTN), a central regulator of the actin cytoskeleton, which disrupts actin organization and leads to decreased proplatelet formation [48]

The biological relevance and functional capacity of MEG-01-derived PLPs for cancer research applications was recently demonstrated by our laboratory in a companion study. Ceron-Hernandez et al. engineered MEG-01 cells to express GFP-tagged oncogenic KRAS^G12D^ and showed that differentiated PLPs carrying this mutation could transfer functional KRAS^G12D^ protein and mRNA to H1975 non-small cell lung cancer (NSCLC) cells, resulting in enhanced tumor cell proliferation, invasion and resistance to tyrosine kinase inhibitors [49]. This transfer confirmed that PLPs can serve as vehicles for horizontal oncogene transfer, recapitulating the tumor–platelet interactions observed in vivo where tumor-educated platelets (TEPs) contribute to metastasis and therapy resistance.

The complexity of EV preparations arises from their inherent heterogeneity, which is further influenced by the specific isolation methods employed. Differential centrifugation, although widely used and compliant with MISEV2023 operational guidelines, is known to co-isolate non-vesicular components such as cellular debris, protein aggregates, and lipoproteins.

EV preparations are intrinsically heterogeneous, with each vesicle containing varying concentrations and combinations of molecules as revealed by the multimodal size distribution for both the MEG-01-derived EXOs and MVs of our NTA. This size-based segregation is a critical feature that highlights the inherent biological heterogeneity of these preparations and aligns with the current scientific consensus [50]. Recent sophisticated multi-omics analyses confirm that physical properties like size and density are primary determinants of an EV’s molecular identity and functional capabilities. Specifically, physically distinct subpopulations possess fundamentally different protein, lipid, and RNA payloads [51]. Furthermore, this molecular divergence translates directly into specialized biological activities [52]. Nevertheless, EV heterogeneity is currently one of the major challenges that has to be addressed by the EV field. The most recent research aims to accurately characterize the physical and molecular properties of EVs at the individual vesicle level. This allows us to know what each vesicle contains and how it differs from the others. However, there is no single perfect technology for this task. For example, AF4-based size fractionation enables the enrichment of distinct EV subpopulations, including large EVs (90–120 nm), small EVs (60–80 nm), and exomers (<50 nm). Biochemical analyses reveal marked compositional and functional differences among these subsets, underscoring the need for advanced high-resolution fractionation platforms and improved methods to map molecular localization across particle sizes [53]. High-resolution fractionation of EVs remains a significant technical challenge, primarily limiting the separation of distinct functional subpopulations based on subtle size differences. [50].

Another critical challenge in this area is the isolation method. Differential centrifugation, although widely used and compliant with MISEV2023 guidelines [20], is known to co-isolate non-vesicular components such as protein aggregates, lipoproteins, and cellular debris. These impurities may influence proteomic profiles and obscure functional interpretation. While measures such as exosome-depleted FBS, sequential filtration, and marker-based validation reduce contamination, complete removal of non-vesicular material is technically difficult. Future studies should incorporate additional purification strategies, such as density gradient ultracentrifugation or immunoaffinity capture, and consider complementary non-vesicular reference fractions to further improve sample purity and refine functional interpretation.

The comparative proteomic analysis of MVs of MEG-01 and those of platelet origin reveals a substantial overlap, with nearly the entire platelet MV proteome represented in the MVs of the MEG-01 dataset. Both vesicle types share biogenic mechanisms, characterized by cytoskeleton-dependent budding regulated by calcium signaling and the Rho/ROCK pathway, resulting in marked structural and functional homogeneity [54]. Functionally, MVs of MEG-01 are enriched in canonical platelet pathways, including PI3K subunit p85 in actin organization and cell migration, platelet activation, signaling, and aggregation, and hemostasis, underscoring their preservation of hemostatic, metabolic, and immunomodulatory features typical of platelet-derived vesicles [2]. Minor variations, such as upregulation of MYC and translation pathways, are attributable to the megakaryocytic origin of MEG-01 cells, but do not result in significant proteomic differences between the two MV types.

These findings establish the MEG-01 cell line as a robust and versatile in vitro model for studying platelet MV biogenesis and molecular mechanisms, as well as facilitating genetic manipulation of MV populations. Moreover, this model serves as an ideal platform for developing therapeutic strategies based on MVs with defined hemostatic and immunomodulatory properties.

The most striking finding of this study was the remarkable proteomic convergence between EXOs of Plasma and EXOs of MEG-01, evidenced by minimal differential pathway activation and near-identical upstream regulator profiles in IPA. This unexpected similarity intersects with an ongoing debate regarding the cellular origins of circulating CD41^+^/CD42b^+^ EVs. While traditionally attributed to activated platelets, accumulating evidence suggests that megakaryocytes directly contribute a substantial fraction through constitutive release mechanisms. Flaumenhaft et al. demonstrated that circulating CD41^+^/CD42b^+^ microparticles in healthy humans are predominantly CD62P^-^ and express full-length filamin A, molecular signatures characteristic of megakaryocyte-derived rather than platelet-derived microparticles. This distinction is critical: CD62P (P-selectin) marks activation-induced vesiculation from mature platelets, whereas its absence indicates megakaryocytic origin through constitutive, non-activated release [55]. Holcar et al. reinforced this complexity, reporting that plasma EVs positive for CD41/CD42b but negative for CD62P likely originate from megakaryocytes, though relative contributions remain debated given the vast numerical disparity between circulating platelets (150–410 × 10^9^/L) and bone marrow megakaryocytes (~0.05% of nucleated cells) [56]. Nevertheless, each megakaryocyte produces 2000–10,000 platelets and directly releases submicron vesicles, establishing the megakaryocytic lineage as one of the most prolific EV-producing cellular systems.

However, this convergence should not be interpreted as definitive enrichment of megakaryocyte-derived vesicles in plasma exosomes. Plasma EVs are highly heterogeneous and originate from multiple cell types, and the fraction analyzed here may be influenced by the isolation protocol and starting material. Other blood-cell-derived EVs may share core hemostatic or cytoskeletal proteins, and our analysis focused on proteomic and pathway-level comparisons rather than comprehensive surface marker profiling beyond CD41/CD42/CD63, which would require additional targeted approaches to fully resolve the cellular origin. Therefore, the observed similarity suggests a substantial contribution of megakaryocyte/platelet lineage EVs to the plasma exosome proteome under these conditions, but additional studies using broader marker panels and orthogonal approaches will be required to confirm lineage-specific enrichment. The proteomic convergence we observed likely reflects that MEG-01 cells, which constitutively produce CD41^+^/CD42b^+^/CD62P^–^ exosomes, share molecular features with megakaryocyte-derived EV populations present in plasma. This similarity suggests that MEG-01-derived exosomes may serve as useful models for subsets of plasma EVs enriched in megakaryocyte/platelet lineage markers, while acknowledging that plasma EVs are heterogeneous and include contributions from multiple cell types.

Beyond biomarker applications, the convergence between MEG-01 derivatives and natural counterparts (platelets, platelet-derived EV and plasma EVs) has immediate therapeutic implications. This work opens multiple avenues for mechanistic investigation of platelets: dissecting molecular determinants of tumor-platelet adhesion using engineered PLPs with mutated or deleted integrins; modeling TEP biology by exposing PLPs to tumor-conditioned medium and profiling transcriptomic/proteomic changes and studying platelet-mediated protection of circulating tumor cells from immune surveillance. Moreover, platelets and platelet-derived EVs are being explored as natural nanocarriers for targeted drug delivery in cancer therapy, leveraging their tumor-homing properties, extended circulation half-life and immune compatibility [57,58]. EXOs of MEG-01 could accelerate this translational pipeline by providing standardized, genetically modifiable starting material for drug loading optimization, surface engineering (peptide display, antibody conjugation) and preclinical efficacy testing in orthotopic tumor models.

Future work should integrate optimized purification methods with enhanced differentiation protocols to generate second-generation MEG-01 derivatives that more faithfully recapitulate native platelet biology. Strategies could include conditions favoring either activated or quiescent phenotypes, depending on experimental needs. For activated states, options include combining VPA with platelet agonists such as thrombin or GPVI ligands to mimic physiological activation cascades. For quiescent states, measures such as calcium depletion during isolation, addition of prostaglandin E1 (PGE1), and optimized buffer composition could help prevent premature activation. Further differentiation refinements may involve thrombopoietin receptor agonists, GATA1 upregulation, or transient MYC suppression to promote terminal maturation without activation. In parallel, single-cell RNA sequencing of differentiating MEG-01 populations could identify transcriptional trajectories and bottlenecks, guiding targeted interventions. Single-vesicle proteomics using advanced mass spectrometry or proximity ligation assays could resolve heterogeneity within EV populations and identify subsets with maximal physiological fidelity. Finally, in vivo biodistribution studies comparing MEG-01 EVs versus native platelet EVs in tumor-bearing mice would validate therapeutic potential and inform clinical translation strategies.

## 5. Conclusions

MEG-01-derived EVs provide important advantages as experimental models, offering a standardized and scalable source with defined proteomic properties that facilitate reproducibility and controlled manipulation. These features make them particularly useful for studies requiring consistent EV preparations and genetic engineering. Our comprehensive proteomic analysis demonstrates strong convergence with megakaryocyte- and platelet-derived EVs in hemostasis-related and cytoskeletal pathways, supporting their use as surrogate models for selected aspects of platelet EV biology. However, they remain surrogates rather than full equivalents of native platelets. Limitations include incomplete terminal megakaryocytic differentiation, persistence of proliferative transcriptional programs, and a predominance of activation-like phenotypes rather than quiescent states, as well as the transformed genotype of MEG-01 cells, which may influence vesicle composition. These conclusions are based on proteomic and pathway enrichment analyses, not on direct functional assays. Future optimization of purification and differentiation protocols could further enhance physiological relevance, positioning MEG-01 derivatives as practical platforms for biomarker discovery, mechanistic research, and exploratory therapeutic applications in oncology and regenerative medicine.

## Data Availability

Raw mass spectrometry data from this experiment have been deposited in the ProteomeXchange Consortium (http://proteomecentral.proteomexchange.org) (accessed on 26 October 2025) through the PRIDE repository [59], with the dataset identifier PXD070221.

## Supplementary Materials

The following supporting information can be downloaded at: https://www.mdpi.com/article/10.3390/biom15121698/s1, Figure S1: Proteomic and Functional Characterization of MEG-01 Cells and their extracellular products. Figure S2: Proteomic and Functional Analysis of Human Platelets (Control and Activated), MEG-01 Cells and PLPs. Figure S3: Proteomic and Functional Analysis of MVs Obtained from Human Platelets (Control and Activated), Plasma, and MEG-01 Cells. Figure S4: Proteomic and Functional Analysis of exosomes (EXOs) Purified from Human Platelets (Control and Activated), Plasma and MEG-01 Cells.

## Abbreviations

The following abbreviations are used in this manuscript:

EVs	Extracellular Vesicles
EXOs	Exosomes
hPSCs	Human Pluripotent Stem Cells
HSCs	Hematopoietic stem cells
LC-MS/MS	Liquid chromatography–mass spectrometry
MVs	Microvesicles
MVBs	Multivesicular Bodies
NTA	Nanoparticle tracking analysis
PLPs	Platelet-like particles
PLTs	Platelets
PRP	Human platelet-rich plasma
TEM	Transmission Electron Microscopy
VPA	Valproic acid
