# Supplementary material for: Proteomic Validation of MEG-01-Derived Extracellular Vesicles as Representative Models for Megakaryocyte- and Platelet-Derived Extracellular Vesicles
Source: Biomolecules. 2025 Dec 5;15(12):1698. doi: 10.3390/biom15121698 (PMC12730508; doi:10.3390/biom15121698)

Suppl. Figure S1 Sanchez-Manas JM *et al.*

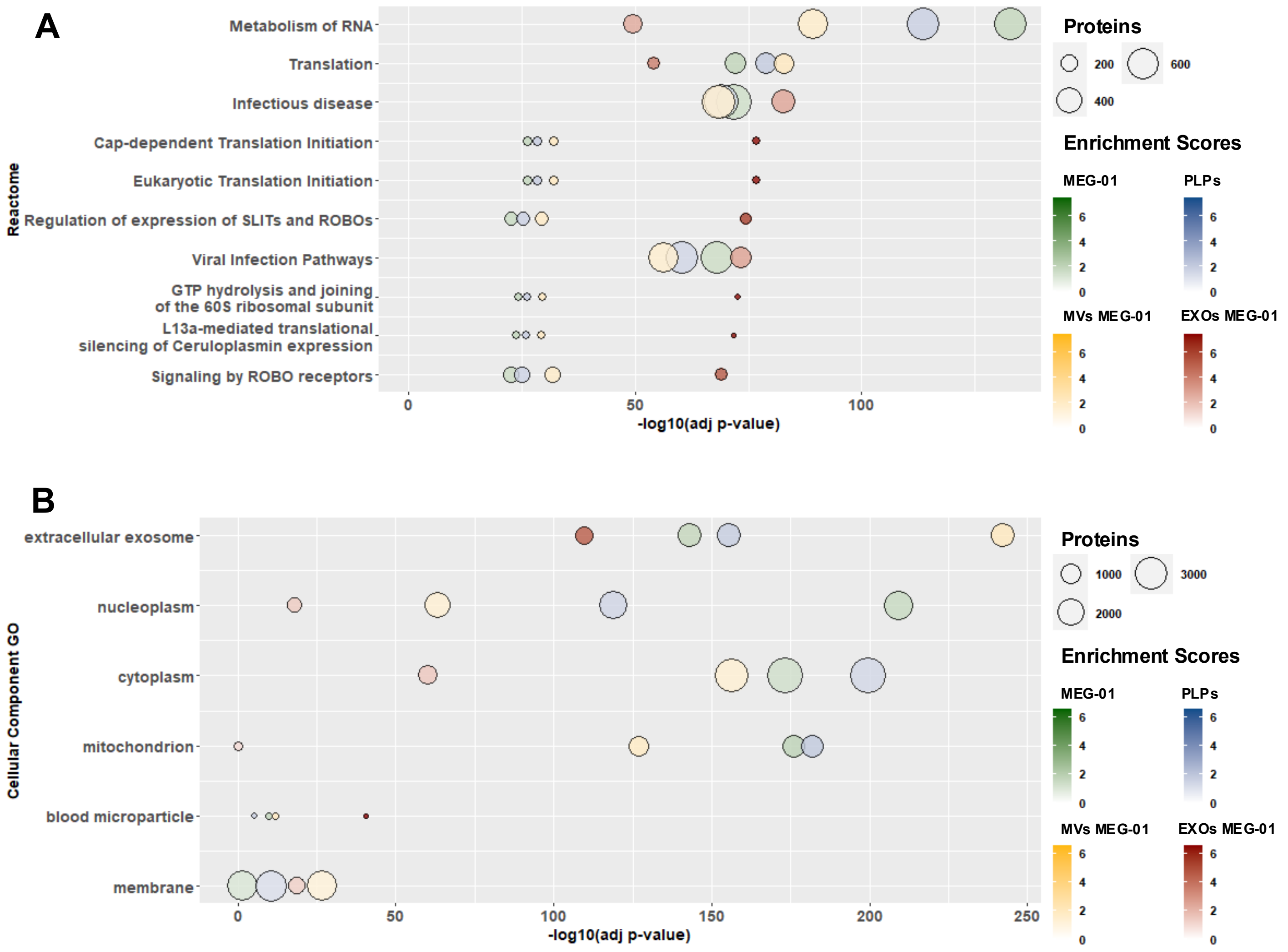

Suppl. Figure S2 Sanchez-Manas JM *et al.*

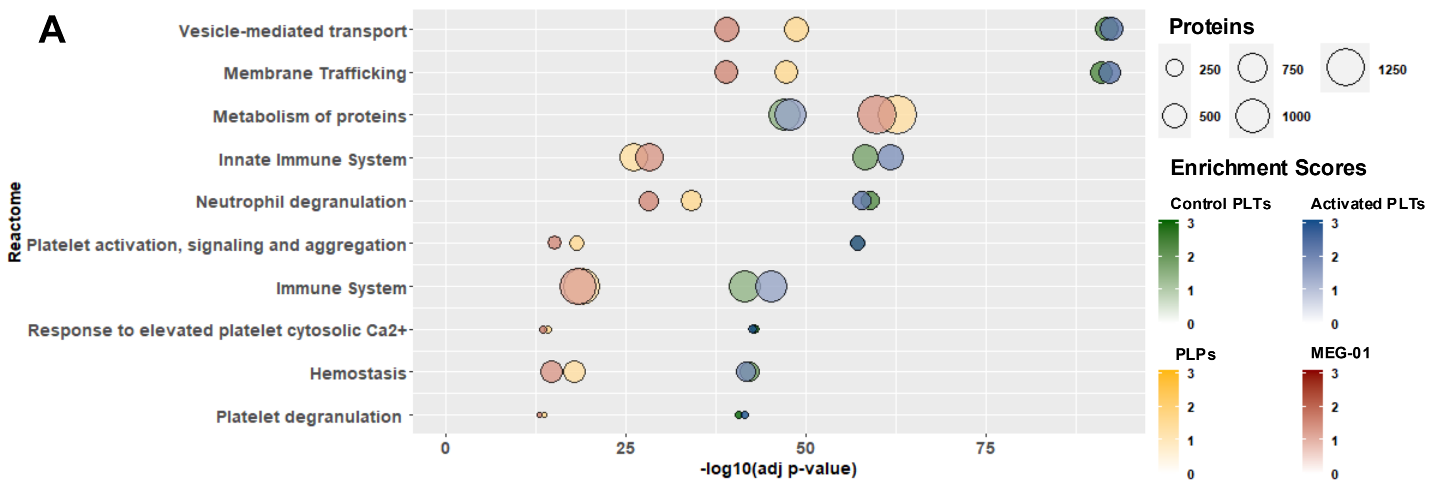

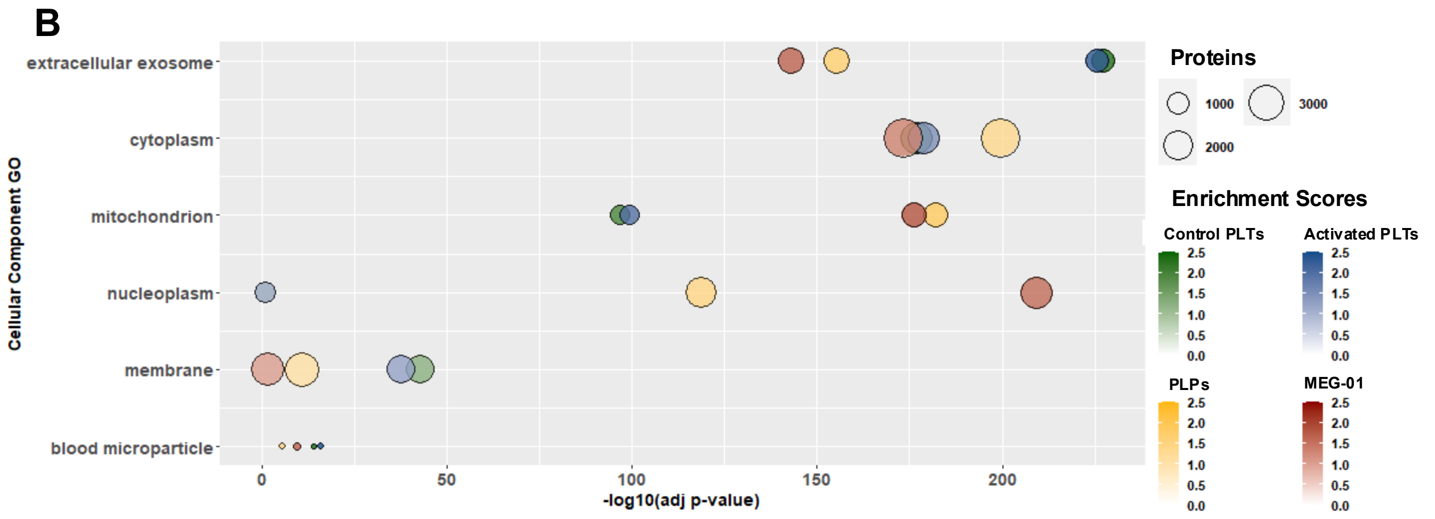

Suppl. Figure S3 Sanchez-Manas JM *et al.*

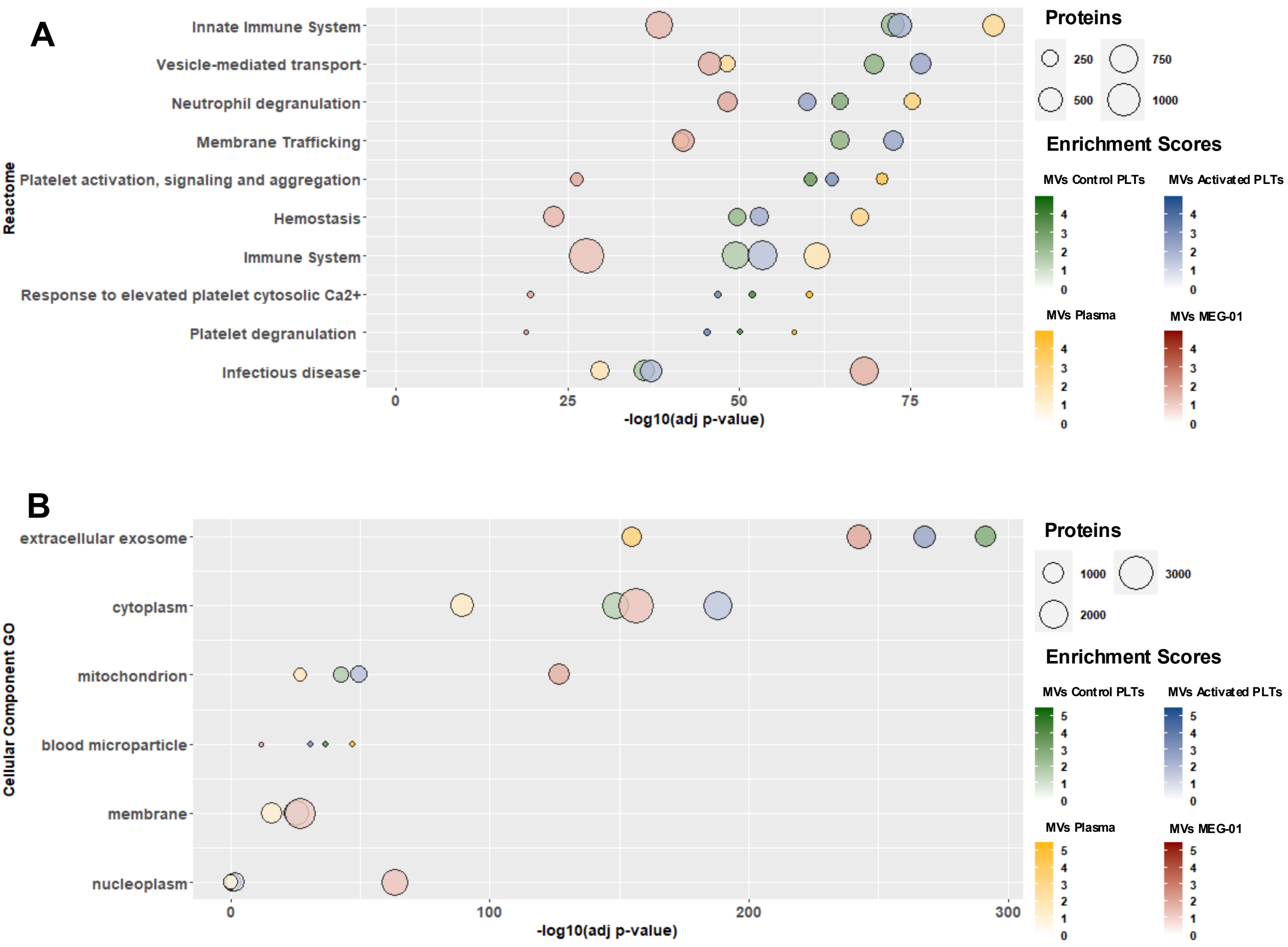

Suppl. Figure S4 Sanchez-Manas JM *et al.*

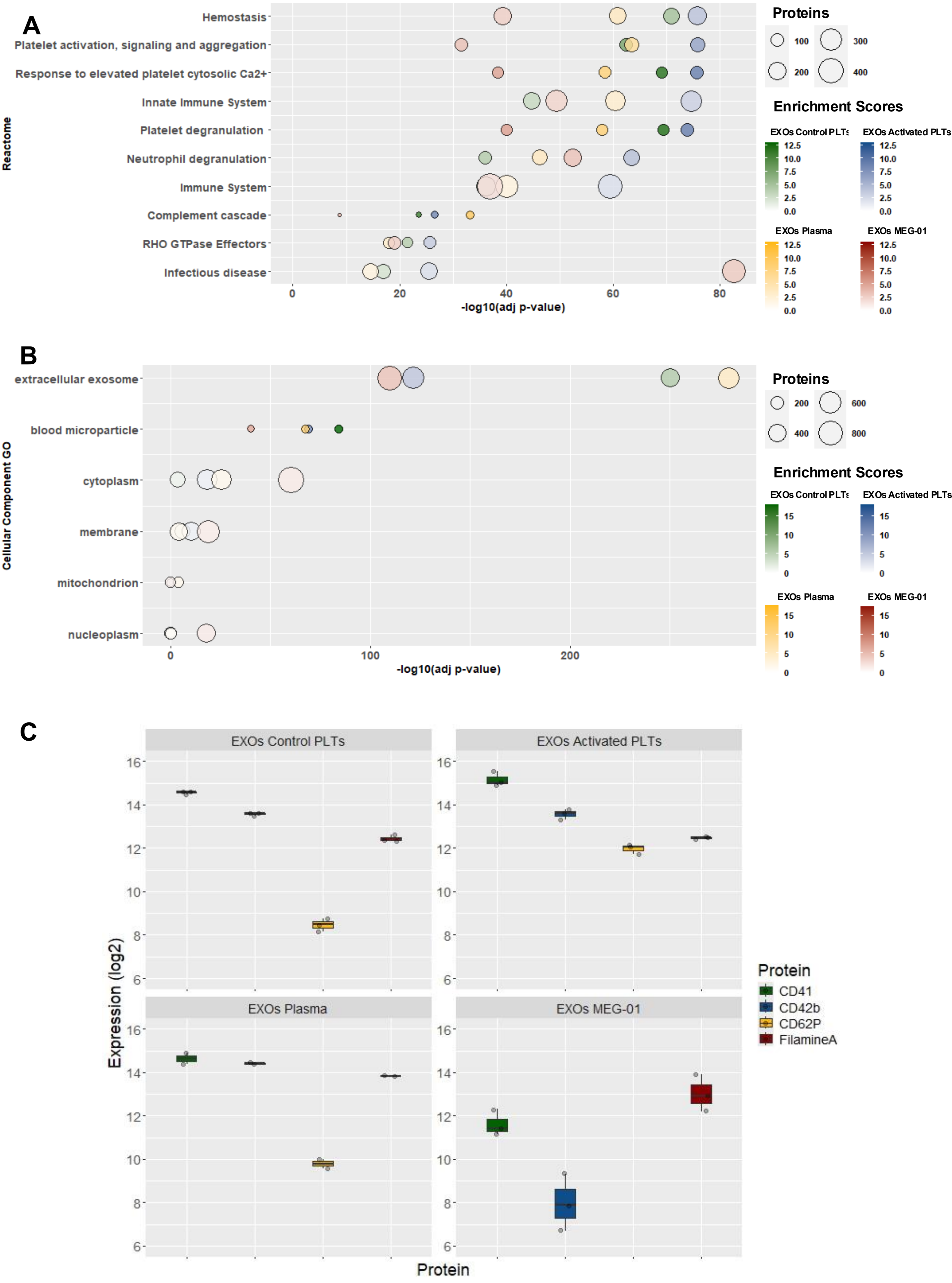

Supplement: Supplementary file 1 [file biomolecules-15-01698-s001.zip › biomolecules-3990991-supplementary.pdf]
